# Insights into the structure and morphogenesis of the giant basal spicule of the glass sponge *Monorhaphis chuni*

**DOI:** 10.1186/s12983-021-00440-x

**Published:** 2021-11-08

**Authors:** Andrzej Pisera, Magdalena Łukowiak, Sylvie Masse, Konstantin Tabachnick, Jane Fromont, Hermann Ehrlich, Marco Bertolino

**Affiliations:** 1 Institute of Paleobiology, Polish Academy of Sciences, ul. Twarda 51/55, 00-818 Warsaw, Poland; 2 Sorbonne Université, CNRS, Laboratoire de Chimie de la Matière Condensée de Paris (LCMCP), 4 place Jussieu, 75005 Paris, France; 3 P.P. Shirshov Institute of Oceanology, Russian Academy of Sciences, 36, Nakhimovski prospect, Moscow, Russia; 4 Western Australian Museum, Locked bag 49, Welshpool DC, WA 6986 Australia; 5 Institute of Electronic and Sensor Materials TU Bergakademie Freiberg, Gustav-Zeuner Str. 309599, Freiberg, Germany; 6 Center for Advanced Technology, Adam Mickiewicz University, 61614 Poznan, Poland; 7 A.R. Environmental Solutions, ICUBE-University of Toronto Mississauga, Mississauga, ON L5L 1C6 Canada; 8 Dipartimento Di Scienze Della Terra Dell’Ambiente E Della Vita (DISTAV), Università Degli Studi Di Genova, Corso Europa, 26, 16132 Genoa, Italy

**Keywords:** Basal spicule, *Monorhaphis chuni*, Sclerocytes, Spicule formation, Spicule structure

## Abstract

**Background:**

A basal spicule of the hexactinellid sponge *Monorhaphis chuni* may reach up to 3 m in length and 10 mm in diameter, an extreme case of large spicule size. Generally, sponge spicules are of scales from micrometers to centimeters. Due to its large size many researchers have described its structure and properties and have proposed it as a model of hexactinellid spicule development. Thorough examination of new material of this basal spicule has revealed numerous inconsistencies between our observations and earlier descriptions. In this work, we present the results of detailed examinations with transmitted light and epifluorescence microscopy, SEM, solid state NMR analysis, FTIR and X-ray analysis and staining of *Monorhaphis chuni* basal spicules of different sizes, collected from a number of deep sea locations, to better understand its structure and function.

**Results:**

Three morphologically/structurally different silica layers i.e. plain glassy layer (PG), tuberculate layer (TL) and annular layer (AL), and an axial cylinder (AC) characterize adult spicules. Young, immature spicules display only plain glassy silica layers which dominate the spicule volume. All three layers i.e. PG, TL and AL can substitute for each other along the surface of the spicule, but equally they are superimposed in older parts of the spicules, with AL being the most external and occurring only in the lower part of the spicules and TL being intermediate between AL and PG. The TL, which is composed of several thinner layers, is formed by a progressive folding of its surface but its microstructure is the same as in the PG layer (glassy silica). The AL differs significantly from the PG and TL in being granular and porous in structure. The TL was found to display positive structures (tubercles), not depressions, as earlier suggested. The apparent perforated and non-perforated bands of the AL are an optical artefact. The new layer type that we called the Ripple Mark Layer (RML) was noted, as well as narrow spikes on the AL ridges, both structures not reported earlier. The interface of the TL and AL, where tubercles fit into depressions of the lower surface of the AL, represent tenon and mortise or dovetail joints, making the spicules more stiff/strong and thus less prone to breaking in the lower part. Early stages of the spicule growth are bidirectional, later growth is unidirectional toward the spicule apex. Growth in thickness proceeds by adding new layers. The spicules are composed of well condensed silica, but the outermost AL is characterized by slightly more condensed silica with less water than the rest. Organics permeating the silica are homogeneous and proteinaceous. The external organic net (most probably collagen) enveloping the basal spicule is a structural element that bounds the sponge body together with the spicule, rather than controlling tubercle formation. Growth of various layers may proceed simultaneously in different locations along the spicule and it is sclerosyncytium that controls formation of silica layers. The growth in spicule length is controlled by extension of the top of the axial filament that is not enclosed by silica and is not involved in further silica deposition. No structures that can be related to sclerocytes (as known in Demospongiae) in *Monorhaphis* were discovered during this study.

**Conclusions:**

Our studies resulted in a new insight into the structure and growth of the basal *Monorhaphis* spicules that contradicts earlier results, and permitted us to propose a new model of this spicule’s formation. Due to its unique structure, associated with its function, the basal spicule of *Monorhaphis chuni* cannot serve as a general model of growth for all hexactinellid spicules.

## Background

Silica biomineralization is one of the main processes for creating skeletal structures in prokaryotes and eukaryotes [1] and silica itself is one of the most widely distributed skeletal biominerals [2–4]. Siliceous skeletons can be found in many groups of organisms including bacteria, diatoms, radiolarians, testate amoebae and plants [5]. Silica also plays a dominant role in skeleton formation in three sponge classes – Demospongiae Sollas, 1885, Homoscleromorpha Bergquist, 1978, and Hexactinellida Schmidt, 1870 (for more about spicules see [6–8] with references).

The structure and process of formation of siliceous sponge spicules has been extensively studied in both demosponges and hexactinellids [6, 7, 9–20], and especially by Müller et al. [21–30], Schröder et al. [31, 32] and Wang et al. [33–40].

In Hexactinellida (glass sponges) the siliceous skeleton consists either of loose, or partially or completely fused, spicules that form a rigid choanosomal (internal) framework [41, 42]. The spicules are always of hexactinic or triaxonic (cubic) symmetries or their derivatives, produced by reduction of the spicule primary rays, or branching, developing spines and specific tips [42]. Spicules in hexactinellids are traditionally differentiated and described as microscleres (micrometers scale) and megascleres (micrometers to centimeters scale) but generally following the tradition [42]. Much larger numerous specialized basal spicules (up to several centimeters in length) anchor some hexactinellids (all Amphidiscophora, some Euplectellidae and some Rossellidae of Hexasterophora) in soft sediments [42, 43]. In the most extreme case, which is found in the hexactinellid *Monorhaphis chuni*, a single basal spicule may reach up to 3 m in length and 10 mm in diameter [26]. *M. chuni* is the only species of the family Monorhaphididae, a group of deep sea hexactinellids [44, 45]. This species lives in muddy substrata of the Indo-West-Pacific region inhabiting depths from 500 to 2000 m [44]. It is characterized by the presence of 14 different types of spicules of sizes between a few micrometers and 50 mm (i.e. tautactines, pentactines and microscleres) and a single giant monaxonic basal spicule that anchors the sponge in soft substrate [9, 34, 44]. The cylindrical, round or oval sponge body with linearly arranged large atrial openings/oscula, envelopes the upper part of the basal spicule with its lower part embedded in the sediment [9, 46].

Due to their large size, the basal spicules of *Monorhaphis* became a model subject for numerous studies [29, 33–36, 38, 39, with references]. These studies investigated the spicules’ composition [25, 27, 28, 35, 36] and formation processes [38, 39] from a mechanical and biochemical perspective [15, 17, 25, 34], as well as enzymatic aspects of spicule formation [26, 30] and processes of silica fusion [9, 29]. The nature of the axial filament was also discussed recently in several papers [47–50]. The results of these investigations have frequently been treated as a general model of hexactinellid spicule formation.

In this paper we provide a systematic and comprehensive study of the basal spicule of *Monorhaphis,* which allowed for evaluation of inconsistencies and contradictory ideas on spicule morphogenesis and formation currently present in the literature. Our new observations and data help to understand the initial growth stage of the basal spicules, and development of the complex structure in its later stages. A new model of basal spicule morphogenesis in *Monorhaphis* is presented.

## Material

**Fig. 1:**
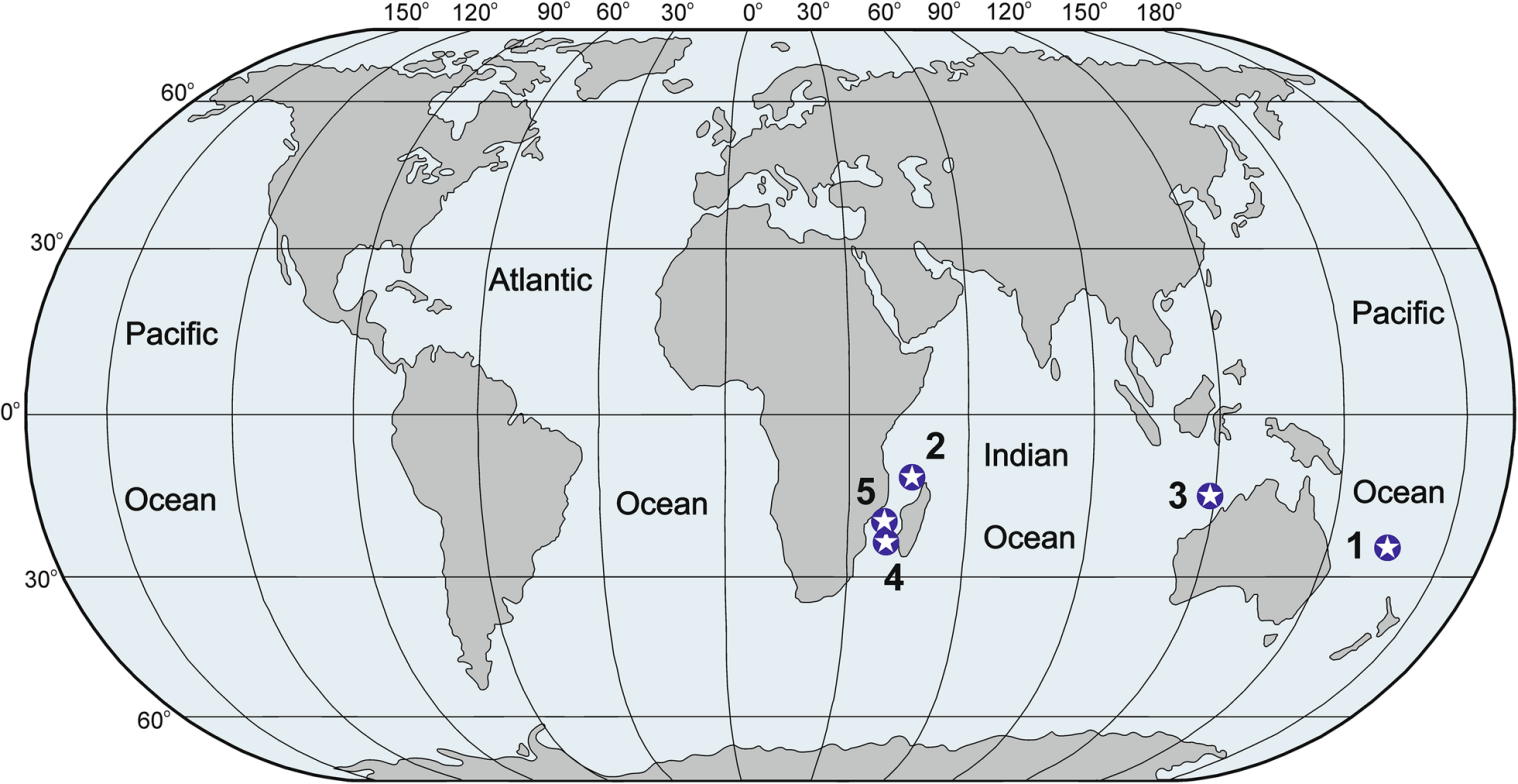
Location of collection of the investigated material of basal spicules of *Monorhaphis chuni,* numbers correspond to numbers in the text

Material for the study consists of several specimens from the following locations (Fig. 1):

1. SW Pacific (New Caledonia, no exact location available, depth about 700 to 1000 m), dry.
2. Mozambique Channel (IORAS 5/2/1352 – ‘Vitjaz II’, 12°31.50′–25.04′S, 48°05.50′–08.00E; depth 700 m), dry.
3. W Indian Ocean (16°38′11″S, 119°08′51″E to 16°38′41″S and 119°08′11″E, WAM Z36164, (fragment) depth 991–988 m; WAM Z36156 Mermaid Reef, CSIRO, RV Southern Surveyor cruise SS0507 2002 16°38′04″S 119°09′13″E to 16°38′46″ and 119°08′02″E, station SS0507/068, depth 983 m), preserved in 60–70% ethanol.
4. E Indian Ocean (Mozambique Channel, canyon facing Tulear, depth about 800 m), dry (collected by fisherman).
5. E Indian Ocean (Mozambique Channel), expedition MAINBAZA, organized by IEO and MNHN 19° 35' S, 36° 48' E 19° 36' S, 36° 47' E 636 m, preserved in ethanol.

## Methods

### SEM and light microscope analysis

Firstly, all spicules were studied under a binocular microscope in reflected light and, when necessary, their surface was photographed to compare with subsequent SEM images. Spicule fragments were cut with a Buehler IsoMet Low Speed saw, and if necessary, cleaned when necessary from an organic sponge mass (that included sponge body fragments adhering to the surface) with the help of 30% hydrogen peroxide. Finally, the spicule fragments were mounted onto SEM stubs and sputtered with platinum.

Light microscopy analysis of spicules was performed with Olympus SZH and NIKON SMZ1270 binocular microscopes (enlargement up to 30X) and epifluorescence NIKON Eclipse Ni microscope. A scanning electron microscope Phillips XL-20 was used to study the detailed morphology of the spicules.

### Solid state NMR analysis

Solid State NMR (ssNMR) measurements were performed with Bruker (Karlsruhe, Germany) Avance III spectrometers at 7.04 T (hereafter called AV300, corresponding to a spin frequency of SF = 300.13 and 75.51 MHz for ^1^H and ^13^C, respectively) and 16.42 T (designated as AV700, operating at SF = 700.14 and 139.10 MHz for ^1^H and ^29^Si, respectively). Samples were packed into ZrO_2_ rotors. Magic Angle Spinning (MAS) was performed at *ca.* 12 kHz of rotation frequency (RO) in a 4 mm rotor on AV300 and at RO = 20 kHz in a 2.5 mm probe on AV700 spectrometer. For ^1^H experiments, OnePulse program was routinely used on AV700 with 4 scans (NS). T_1_ longitudinal relaxation time was checked and a recycle delay (RD) of 5 s appeared to be suitable for quantitative experiments. A spin echo experiment [51] was also undertaken with 1 rotor turn, in order to check proton mobility. Spinal64 High-Power proton Decoupling (ν_1H_ = 71.4 kHz) [52] combined with MAS (HPDec-MAS) was used during acquisition for ^29^Si experiments on AV700, with RD = 60 s at 30° pulse angle to allow for collection of quantitative data, for NS = 4.000 scans. Cross-Polarization (CP-MAS) was used for ^13^C experiments on AV300 with RD = 1 s and 1 ms cross-polarization time (t_CP_), with proton decoupling during acquisition (ν_1H_ = 52.6 kHz) for NS = 976.896 scans, that is to say *ca.*11.5 days acquisition. Probe signal on ^13^C CP spectrum was suppressed using DEPTH pulse sequence [53] placed after the CP transfer step.

During 1D-processing, a line broadening (LB) of 0 Hz, 50 Hz or 100 Hz was applied during Fourier Transform for the ^1^H, ^13^C and ^29^Si spectra, respectively. Chemical shifts (δ) were referenced to tetramethylsilane (TMS; δ = 0 ppm) for all experiments. Experimental quantitative ^29^Si HPDec-MAS spectra were fitted using DmFit modeling software [54]. The deconvolution was made on a spectrum recorded after NS = 4.000 scans for the PG and 6.000 scans for the AL (as there is only a small amount of powder in the case of the AL, only enough to fill the 2.5 mm diameter rotor to approximately two thirds). The ranges of ^29^Si chemical shifts in solid silicates are classified according to a Q_n_ notation [55] where Q represents the SiO_4_^4−^ units and *n* the degree of connectivity of these units (Table 1).

**Table 1:** The ranges of ^29^Si chemical shifts in solid silicates, derived from [55]

Type of silicates	Q_n_ species	Range of chemical shift (ppm)
Monosilicates	Q_0_	− 66 to − 74
Disilicates and chain end groups	Q_1_	− 75 to − 82
Chain middle groups	Q_2_	− 85 to − 89
Chain branching sites	Q_3_	− 95 to − 100
Three-dimensional framework	Q_4_	− 103 to − 115

The silica condensation degree, so called D, that represents the ratio of Si–O–Si bonds over all Si–O–X bonds (X = H or Si in the present case with only Q species), was calculated using the following formula $D = \frac{{\sum\nolimits_{n = 0}^{4} {nQ_{n} } }}{{4\sum\nolimits_{n = 0}^{4} {Q_{n} } }}$ [55]. The average degree of connectivity $\overline{n}$, that can be expressed by the related formula $\overline{n} = \frac{{\sum\nolimits_{n = 0}^{4} {nQ_{n} } }}{{\sum\nolimits_{n = 0}^{4} {Q_{n} } }}$ [56], was also used in order to better elucidate the 3D silica network.

### X-ray analysis

X-ray diffractograms (XRD) were recorded on a D8 ADVANCE powder diffractometer (Bruker AXS, GmbH, Karlsruhe, Germany), equipped with a Cu source (doublet CuK_α_ wavelength at 1.54056 Å and 1.54439 Å), a Bragg–Brentano θ – 2θ scan and a LynEye linear detector. Experiments were undertaken with a variable slit V6, a step size of 0.009° and 950 s.point^−1^ during ca. 12 h total acquisition, ranging from 5° to 80° (2θ).

Due to a very low amount of powder (only 11 mg for the AL), and also in order to get a higher precision on the minor peaks, XRD experiments were undertaken using a low background silicon sample holder. The two samples of powder were weighed equally in order to compare signal intensities in a quantitative way. A background recording was done to distinguish precisely between baseline, amorphous and crystalline phases.

### FTIR analysis

Infrared spectra of the ground powders were recorded using a Perkin-Elmer Spectrum 400 FTIR spectrometer equipped with an ATR (Attenuated Total Reflectance) sampling accessory provided with a diamond crystal. Spectra show the variation of transmittance (% T) Vs. wavenumbers from 550 to 4000 cm^−1^ with a resolution of 4 cm^−1^.

## Results

### SEM and light microscope analysis

#### Gross spicule morphology

**Fig. 2:**
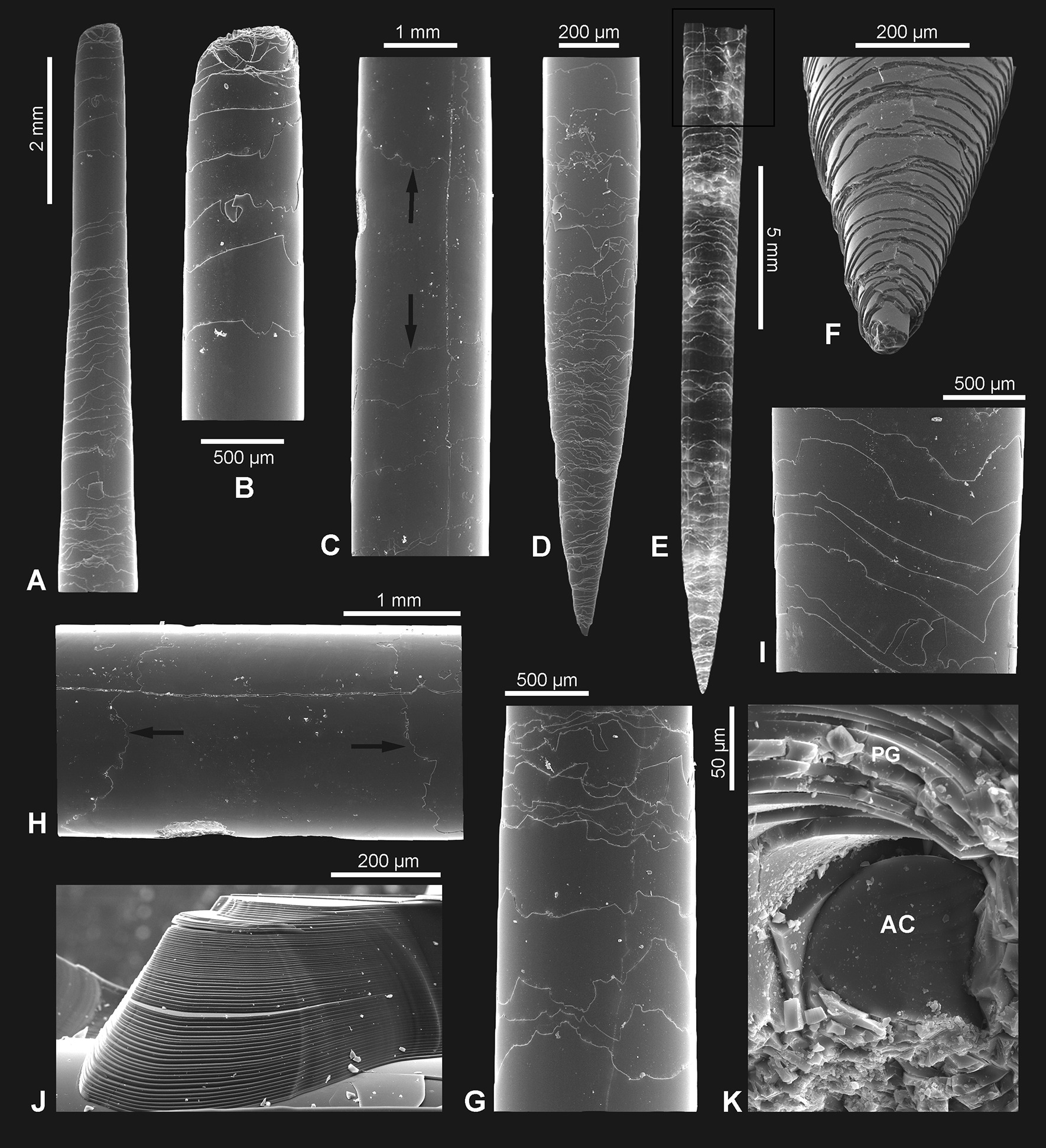
Morphological features of a small (young) *Monorhaphis* spicule as seen with SEM (**a–d, f–k**) and reflected light (**e**). **a**, **b** Apex of the spicule; step-like faces of the plain glassy layer (PG) lamellae are facing toward apex. **c**, **h** Central portion of the spicule; step-like faces of PG lamellae directed in opposite directions are arrowed; details of the same fragment (**h**). **d–f** Lower tip of the spicule showing step-like faces of PG lamellae directed toward the tip; details of the same spicule lower tip shape (**f**). **g** Step-like surfaces of the superimposed layers are facing upward and are mostly conforming in shape. **i** Details of superimposed layers with step-like face that conform in shape. **j** Regular layering (lamellae) of PG in longitudinal broken section. **k** Broken transverse section near the spicule upper tip showing axial cylinder (AC) and surrounding lamellae of PG

All investigated basal spicules that are more or less complete show that the spicules taper toward both ends (Fig. 2a, b, d–f). However, the tapering is not equal and proceeds more gradually toward the apical tip (Fig. 2a, b). The largest complete spicule diameter is at approximately 1/3 of the length. For example, in a spicule that is 133 cm long the maximum thickness is 3.34 mm, 73 cm from the spicule apex. This was also found to apply to incomplete spicules (with the lower end truncated). Two incomplete spicules, which are 134 and 130 cm long respectively, have a maximum diameter of 4 and 4.1 mm, 89 and 92 cm from the spicule apices.

**Fig. 3:**
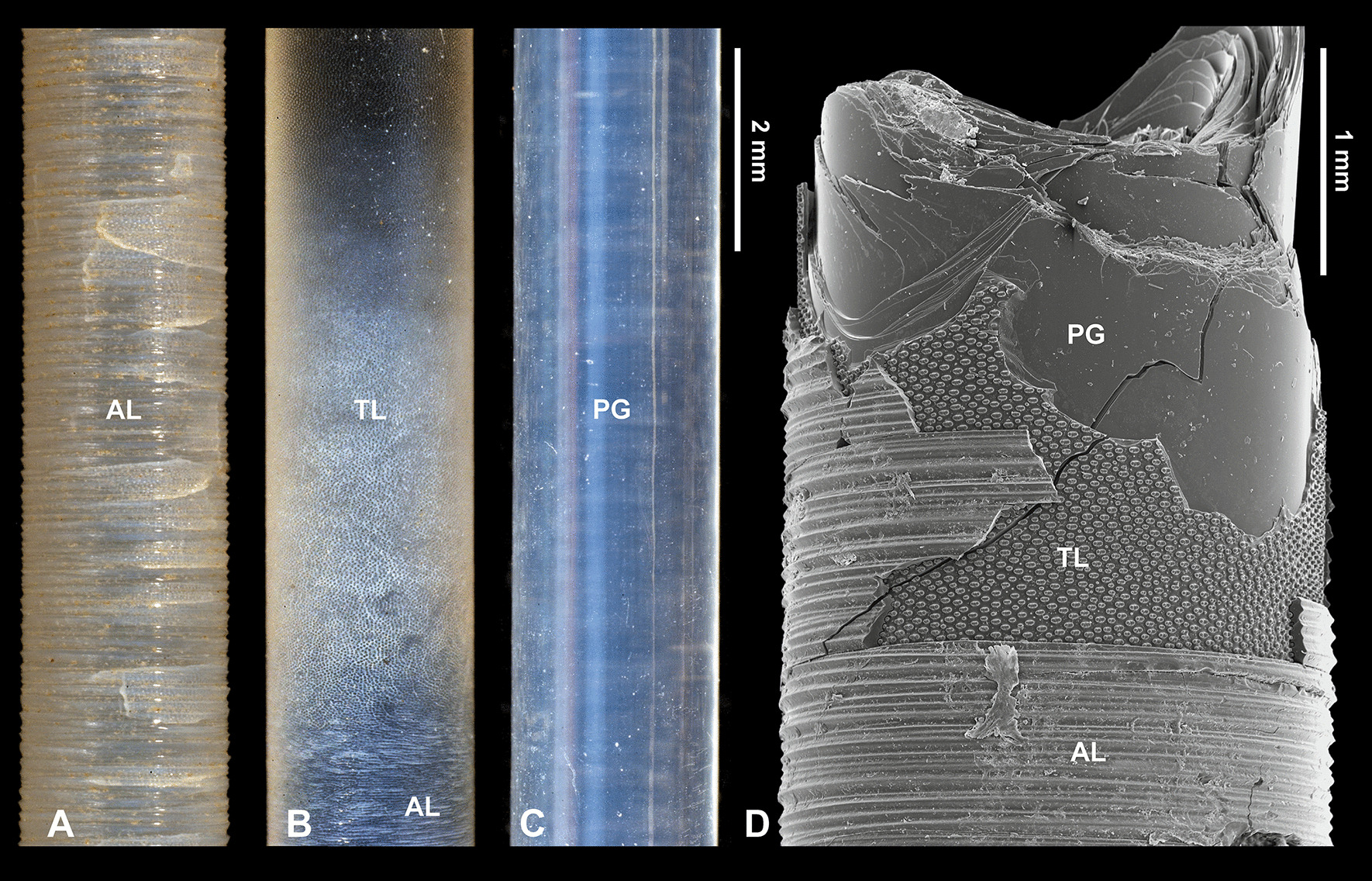
Morphological features of a *Monorhaphis* spicule along its length in reflected light (**a–c**) and with SEM (**d**). **a** Lower part of the spicule displaying the annular surface (AL). **b** Intermediate region showing annular (AL) and tuberculate layers (TL). **c** Upper portion of the spicule showing the plain glassy surface (PG). **d** Broken lower portion of a spicule showing all three structural units superimposed

Mature spicules display three morphological zones along their length. At the base is the annular zone (AL; Fig. 3a), followed by the tuberculated zone (TL; Fig. 3b), and then the plain glassy silica zone (PG; Fig. 3c). The PG zone dominates the spicule length (from the apex, comprising 39 cm in a 50-cm-long spicule) and progresses into the TL zone which is of variable length. The TL zone passes into the AL zone in the lower part of the spicule. However, in a cross section of a lower part of a spicule, all three zones are observed as superimposed layers (Fig. 3d). The PG layer is the most internal, the TL layer is in the middle, and the AL layer is the most external (Fig. 3d). This situation and sequence of morphological zones along the spicule is a natural one as it was observed in a complete sponge with organic mass preserved over the basal spicule.

#### Immature/young spicule

**Fig. 4:**
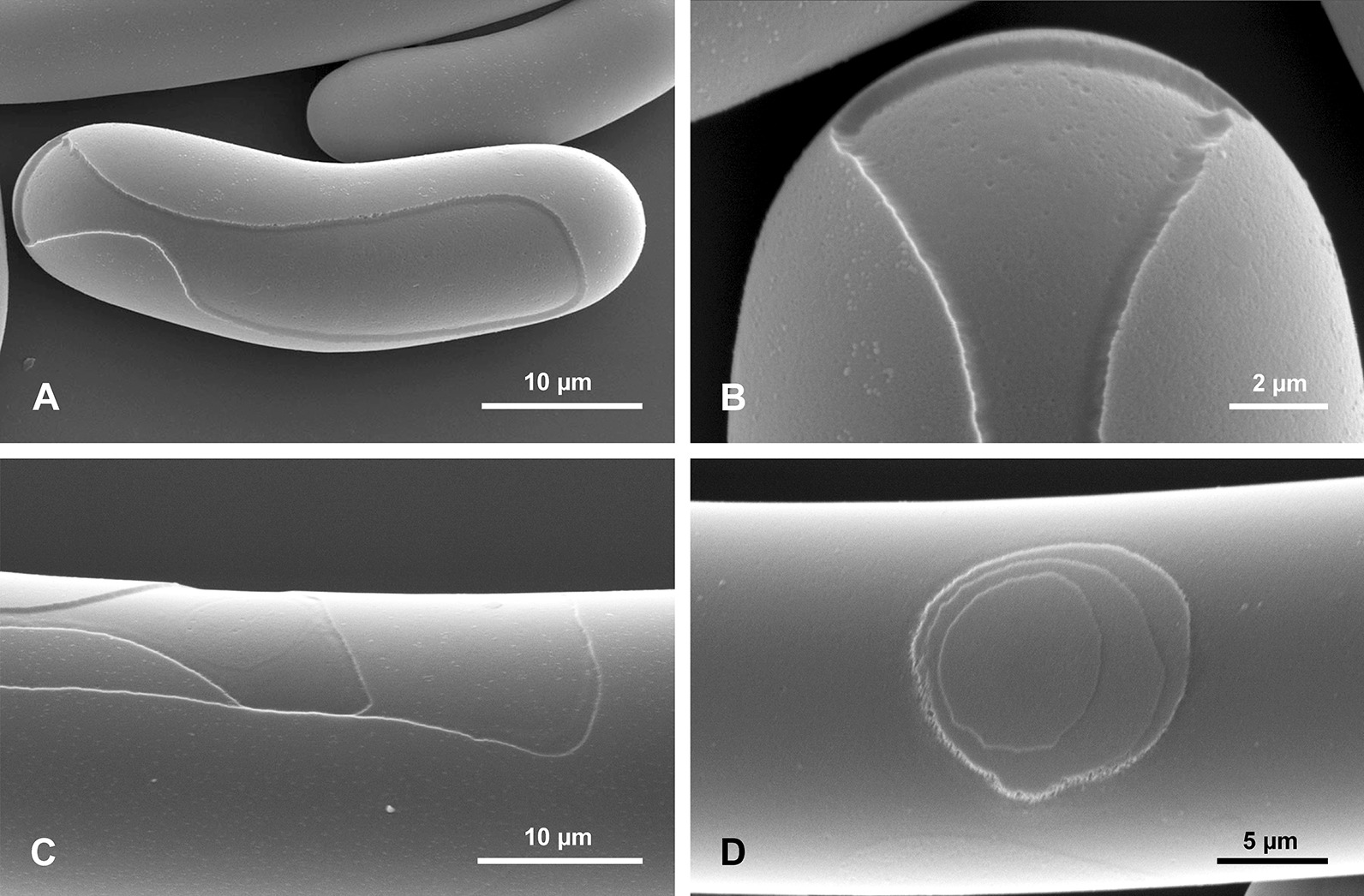
Surface of pristine spicules (extracted from the sponge that was alive when collected) of petrosid desmosponge. **a** Spicule with uncomplete layer of silica showing natural steep faces of newly deposited silica layer; **b** details of **a** to show vertical surface limiting the newly deposited silica layer. **c** Another spicule surface with two superimposed uncomplete layers of newly deposited silica limited by steep faces.** d** Surface of another spicules with superimposed three new incipient layers of silica deposited on the earlier surface of the spicule that are limited by steep faces

In a small, 35-cm-long, immature well preserved spicule, both tips differ from each other. The apical tip is very narrow conical, tapering more gradually (Fig. 2a, b) than the lower tip that is more steeply conical (Fig. 2d–f). This immature spicule differs from the larger specimens by displaying only PG surface layers along the whole length, which seems more or less an original feature not a taphonomic effect. Close examination of the surface of this spicule revealed the presence of numerous patches of silica (incipient layers) (Fig. 2c, h) that are not continuous along the whole spicule length (do not cover the entire spicule). The most external lamellae have a smaller extension along the spicule than the more internal ones (Fig. 2c, h). Moreover, the incipient lamellae only sometimes form patches, occurring on one side of the spicule, and are limited by steep faces (Fig. 2g, i). These faces do not appear to be only fracture features (that dominate), because similar steep faces have been observed in demosponge spicules that were pristine (extracted from a living sponge) (Fig. 4). Often these step-like faces that limit some incipient lamella, conform in a smooth contour shape (Fig. 2g, i), suggesting this can be a natural feature and interpreted as corresponding/following more or less to growth lines, but even those that are artificial fractures and in majority, follow in general original depositing pattern of particular lamellae. Others that display a contour shape with sharp angles are definitely not natural but preservational features, and have resulted from breakage of delicate outer layers. In all cases these steep faces of lamellae are facing the apex in the upper part of the spicules, and the lower tip in the lower part of the spicule (Fig. 2h).

#### Mature spicules

**Fig. 5:**
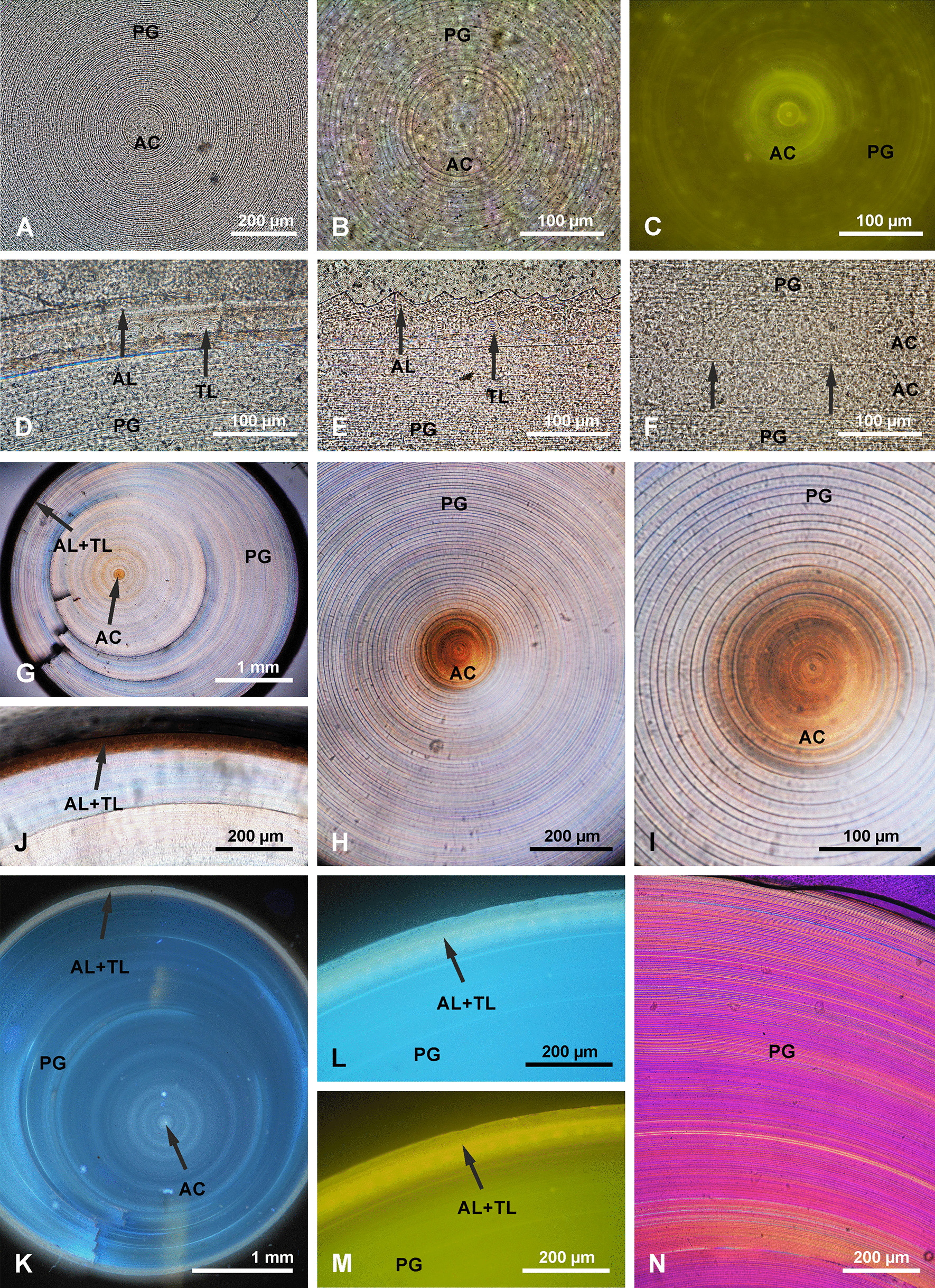
*Monorhaphis* basal spicule in transverse and longitudinal thin (**a–f**) and thick (**g–n**) sections. **a**, **b** Central part of the spicule in transmitted light with regular layering of the plain glassy layer (PG) zone and apparently non-layered axial cylinder (AC); transverse section. **c** Central part of the spicule in green epifluorescent light; layered axial cylinder displays stronger fluorescence; transverse section. **d** Marginal part of the spicules showing AL, TL, and PG zones superimposed (transverse section), transmitted light. **e** Longitudinal thin section showing AL, TL and PG layers superimposed, transmitted light. **f** Longitudinal thin section showing axial canal (arrowed), axial cylinder AC and PG zone. **g–n** Thick transverse sections: **g** showing that most of the spicule consists of PG layers, and asymmetry of spicule; **h**, **i** showing conspicuous layering of PG and that AC is also layered but less regularly; note brown hue (organics) of AC, and that transition from AC into PG zone is clearly continuous (**i**). **j** Section of the marginal zone of the spicule to show dark (high organics content) AL and TL layers. **k** Blue epifluorescence light showing various layers with epifluorescence of different intensity. **l**, **m** Details of marginal zone of the spicules in blue (**l**) and green (**m**) epifluorescence. **n** Crossed polarized transmitted light; note heterogeneity of apparently homogeneous PG zone

In cross section, in transmitted light, the most conspicuous feature of mature spicules is regular layering (also seen with SEM) formed by concentric (about um 4.8–7.4 µm thick) lamellae around a solid axial cylinder (Figs. 2j, k, 5a–c, h, i). In cross section the axial cylinder is clearly delineated from the surrounding PG layers (Figs. 2k, 5a, b, f) at any particular point. However, the first “glassy” layers that occur just beyond the axial cylinder in the direction of the spicule apex merge with the axial cylinder, with each successive layer transgressing over the previous layers in the direction of the apex (not able to be illustrated because it is gradual).

It is interesting to note that some lamellae are thicker on one side then on the opposite side [cf. 9, 17], suggesting zones of asymmetrical growth (Fig. 5g, k). Such asymmetry of the thickness of the lamellae can be observed both in the central zone as well as in the more external zone. The outermost AL is clearly different in structure from the rest of the spicule (which is glassy translucent), being very brown and non-homogenous (suggesting high organic content) (Fig. 5d, e, j, g). Brown coloration also characterizes the axial cylinder (Fig. 5g–i). A lighter brown hue can be observed further from the axial cylinder in the internal regularly layered zone of the spicule (Fig. 5i). With epifluorescence microscopy (green and blue filters) zones of different intensity are clearly visible (Fig. 5k–m), that do not mimic the structural (mineral) layering but appear to correspond to the supposed organic content (i.e. the brown hue described above). The complexity of particular structural layers visible in simple transmitted light is also revealed in crossed polarized light (Fig. 5n).

#### Plain glassy silica layer PG

PG layers comprise most of the spicule volume, but are only visible superficially in the middle and upper portion of the mature spicule; in lower parts being covered first by the TL and then by the AL (Fig. 3c, d). At higher magnification, the spicule surface is smooth and shows no surficial structural elements (Fig. 3d). The PG layers vary in thickness between (2.1 exceptionally) 4.8 µm and 7.4 µm (in most cases), and in the thickest spicules there may be several hundred layers depending on their diameter. In transmitted light it is apparent that it is the translucent PG layers that look structureless when viewed with SEM. In immature spicules only the PG layers are present (see above).

#### Tuberculate layer TL

**Fig. 6:**
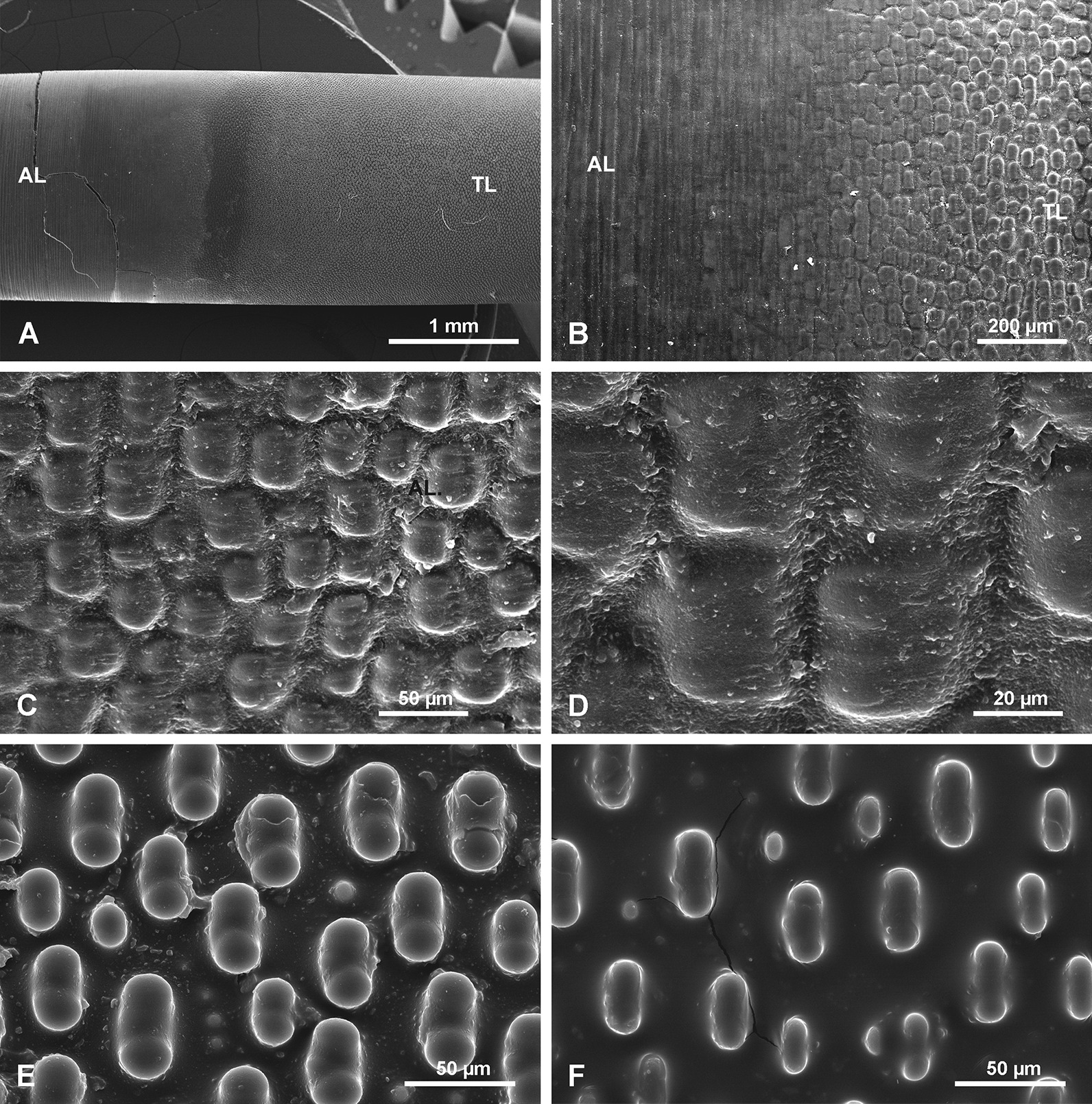
Tuberculate layer surface. **a** General view of the transition from the annular layer surface (AL) to the tuberculate layer surface (TL). **b** Details of the transition between the AL and TL. **c**, **d** Details of tubercles in the transition zone between AL and TL surfaces; (note the irregular shape of tubercles that nearly fuse and that have different morphology from those occurring further along the spicule); **e** Typical, well-developed tubercles further from the annular surface (toward the apex of the spicule). **f** Typical tubercles in early stage of formation (young) on the same surface but located more apically

**Fig. 7:**
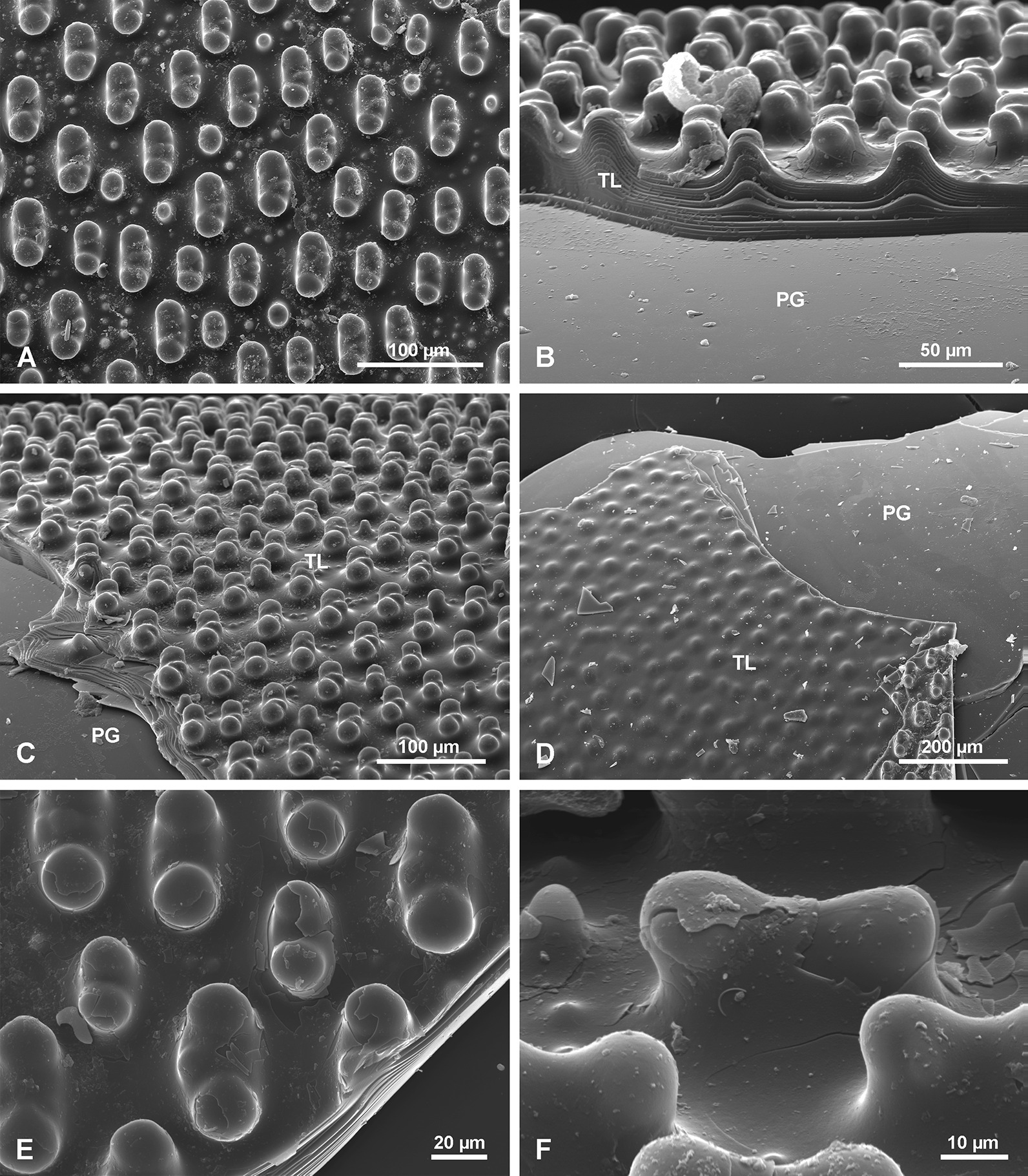
Details of tuberculate layer (TL) surface in top view and in section. **a** Well-developed tubercles, close to the AL surface (note complex character of tubercles and small tubercles occurring in between the main tubercles). **b** Tubercles in cross section showing the transition from the PG surface to the fully formed TL layer. **c** Tuberculate surface in oblique view to show morphology of tubercles. **d** Transition from PG layer to low (incipient) tubercles in the TL

In the more apical part of the TL (Figs. 6, 7) the tubercles first appear as small and low, simply rounded or oval elevations (Fig. 7d) that gradually grow in size towards the lower region where they have more complex shapes (Fig. 6e, f). Incipient and fully developed tubercles occur along the same surface, only in different locations. In cross section there is no structural difference between the TL (that is composed of several sublayers) and the PG layers occurring beneath it. The tubercles are formed by progressive folding of the PG surface (Fig. 7b). The “growing” of tubercles is achieved by the addition of new, more folded sublayers (Fig. 7b–d). The fully developed tubercles are high, elongated, oval in shape (with longer axis horizontal) with a saddle in the middle of the upper surface (Figs. 6e, 7a, c). The fully developed tubercles are 42.0–57.9 µm in length × 23.2 to 29.4 µm in width, and up to 22.1 to 25.8 µm in height. They are very regularly distributed over the spicule surface (Figs. 6e, 7a, c). Sometimes between the fully-developed tubercles, smaller, round tubercles appear (Figs. 6e, f, 7a). Along the spicule surface the transition from the TL to the AL is gradual (Fig. 6a, b); the tubercles become more and more diffuse (caused by the addition of new material in the incipient AL), less regular and more dense and gradually pass into the AL (Fig. 6b–d).

#### Annular layer AL

**Fig. 8:**
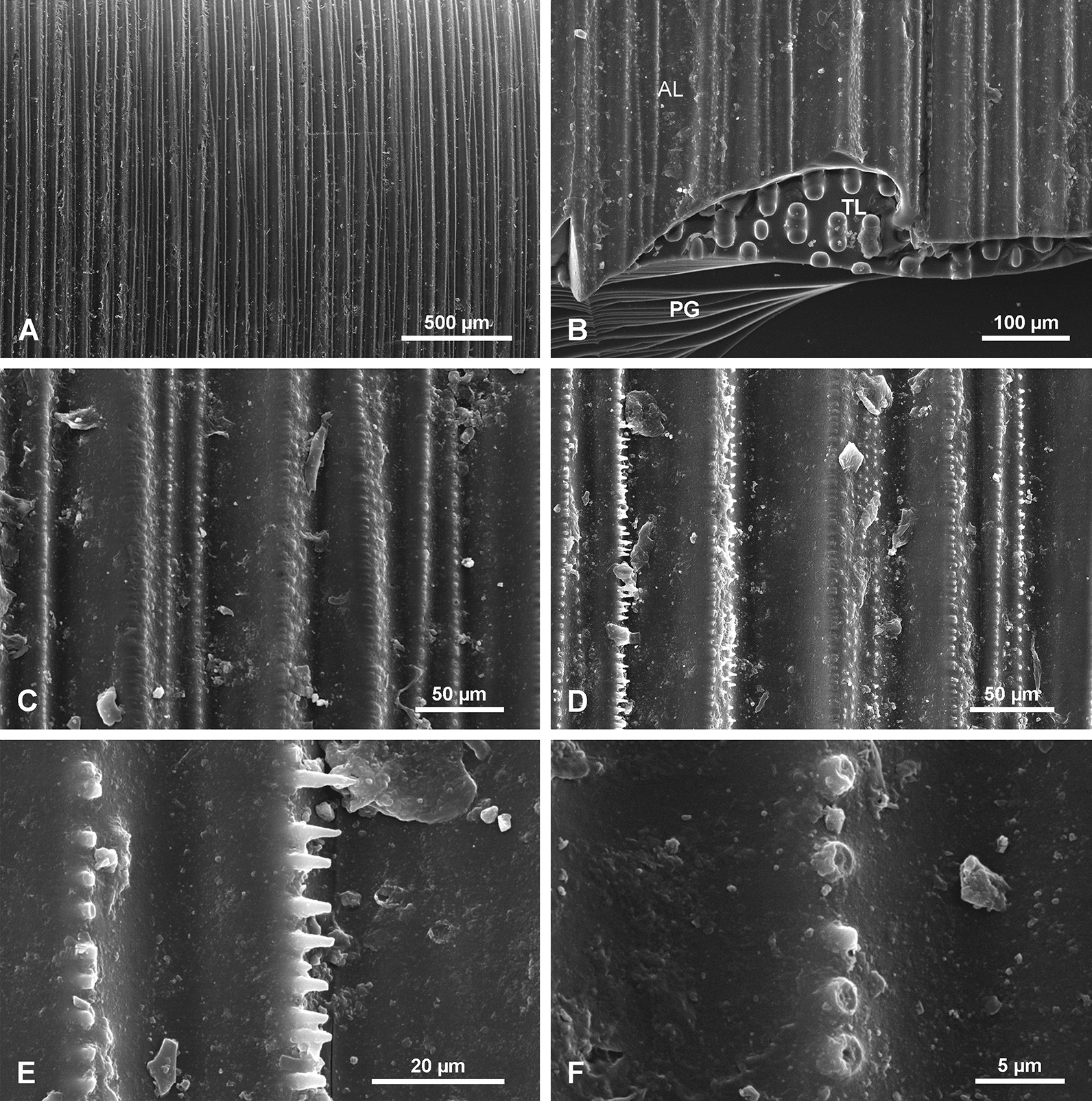
Annular layer (AL) surface. **a** General view; note that some ridges split or branch. **b** Details of ridges that are tuberculate and intercalating with lower ridges. **c** Details of the AL surface showing spinose ridges. **d** Details of broken spines as in c, to show that they are empty internally

The outermost AL has a surface characterized by regularly developed transverse (horizontal) steep and sharp asymmetrical ridges (Fig. 8a, b). Their steeper slopes face the apex of the spicule. Lower ridges are interspersed between the higher ridges (Fig. 8a, b). The annular ridges may branch or fuse, thus not each ridge is continuous around the spicule. The edges of some of the ridges have small (up to 10 µm long), well defined tubercles and narrow spikes directed upward toward the apex of the spicule (Fig. 8c, d). Broken spikes reveal that they are hollow internally (Fig. 8d).

**Fig. 9:**
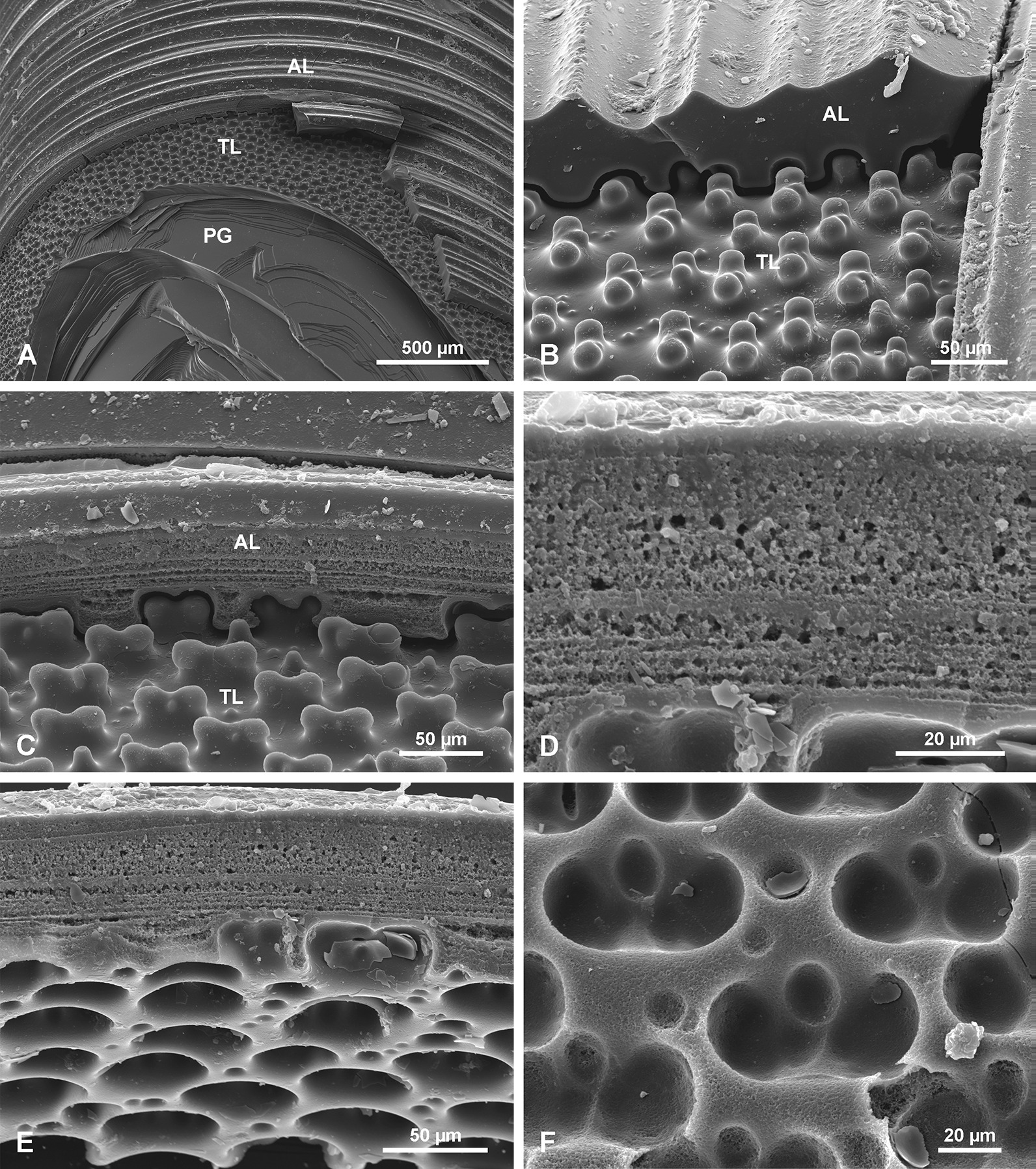
Structure of the annular layer (AL) and its relation to the underlying tuberculate layer (TL) and plain glassy silica (PG). **a** General view of all 3 zones in the spicule: PG, TL and AL. **b**, **c** Contact between AL and underlying TL (note that tubercles fit perfectly into depressions in the lower surface of the AL), it resembles tenon and mortise or dovetail joints. **d** Granular characters of silica in the AL (note layering). **e**, **f** Details of the AL lower surface showing complex regular depressions into which fit tubercles from the underlying layer

A transverse section of the AL reveals that it has a different structure to the rest of the spicule (Fig. 5d, e, j, k, m). The AL is porous and composed of granular silica (Fig. 9c–e). In thick section, canals running obliquely are visible and form the tops of ridges (Fig. 5e). These are probably the same canals that were observed as internal hollows or channels in broken spikes on the ridges.

The lower surface of the AL is not even. It has depressions (Fig. 9b, c, e, f) into which tubercles of the underlying surface fit perfectly (Fig. 9b, e, f). In other words, these depressions in the lower surface of the AL are perfect negatives of tubercles of the TL surface (Fig. 9f). This relationship resembles mortise and tenon, or dovetail joints in woodworking.

**Fig. 10:**
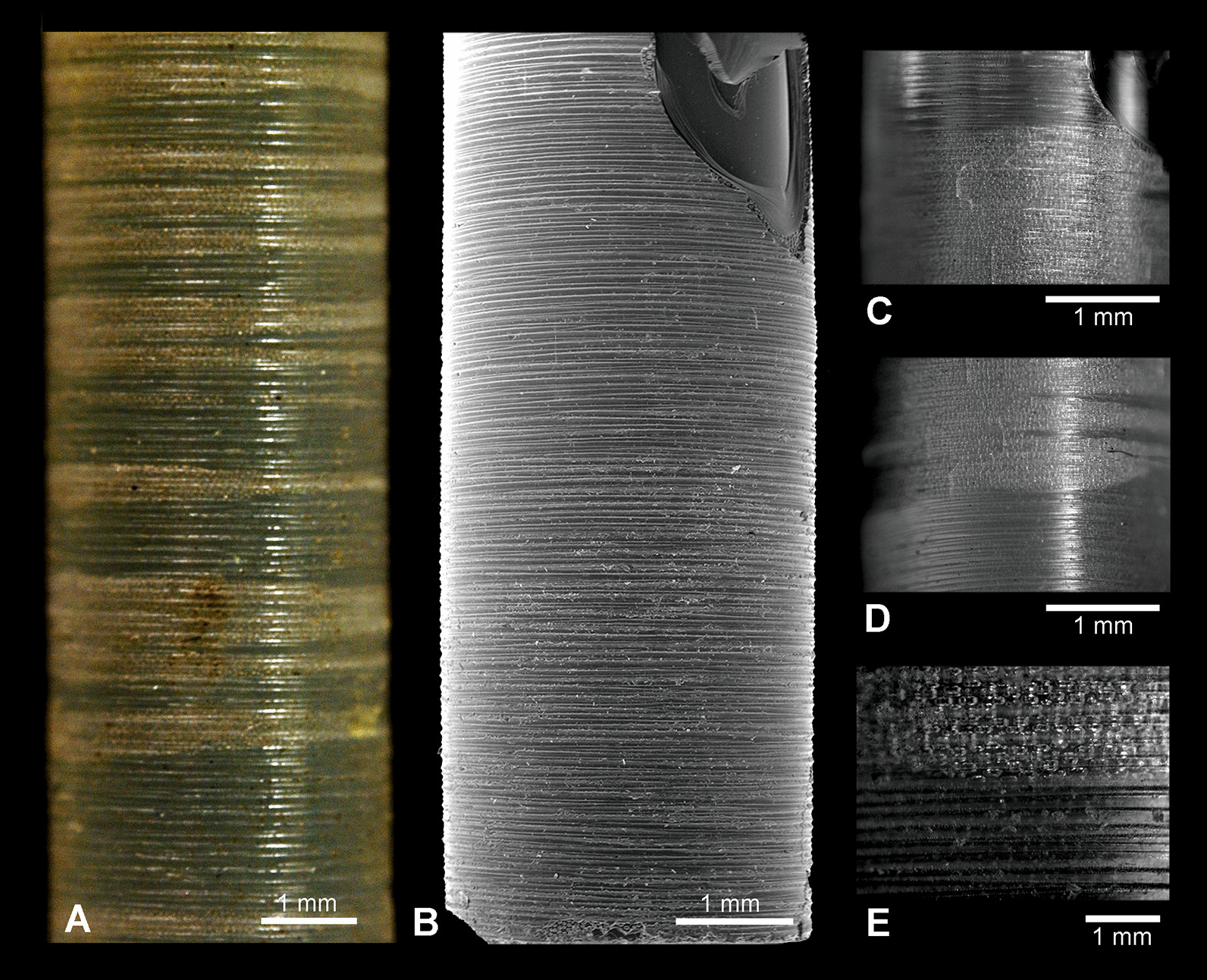
Details of the same annular layer (AL) in reflected light (**a**) and SEM (**b**) to show that the bands observed under reflected light (**a**) are neither regular, nor regularly distributed, are not visible with SEM (**b**). **c–e** Details of various fragments of **b** (**c** corresponds to the top of (**b**) and that supposed “depressions” in the surface of the AL are not visible in SEM, thus that they are optical artefacts

In reflected light many spicules have AL surface zones or bands of different colors (milky and dark grey) that are not visible with SEM (Fig. 10). These bands are irregularly distributed, have various widths along the spicule (there is no pattern in their organization) and the width of each band may vary around the circumference of the spicule (Fig. 10a, d). Those bands that are lighter (milky) can be easily detached from the underlying TL, and the tubercles are clearly visible through them (Fig. 10c–e), while darker bands are very difficult to detach and the underlying tubercles are not visible.

#### Ripple mark surface/layers RML

**Fig. 11:**
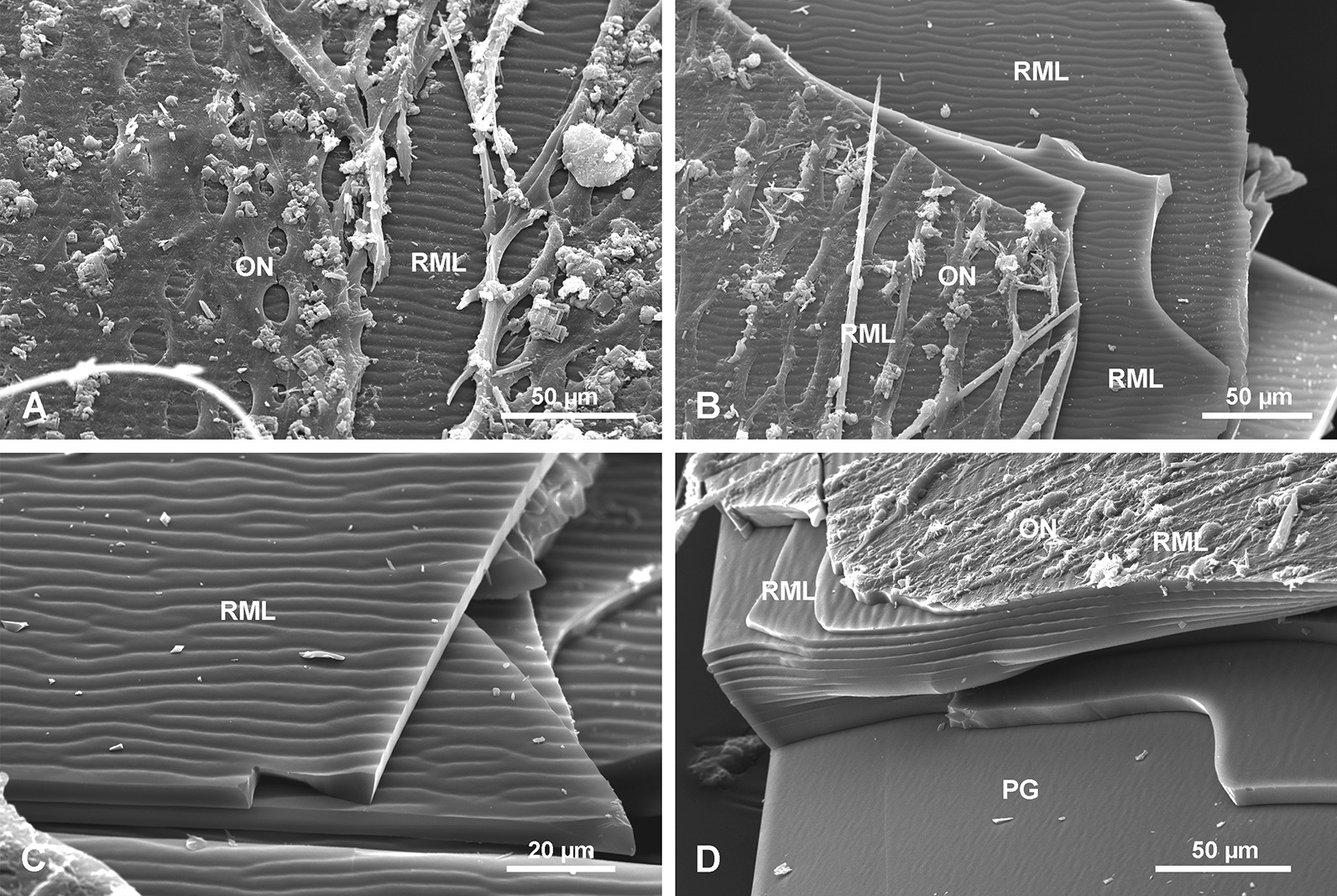
Ripple mark structures (RML) on outer layers of the spicule. **a**, **b** Organic net (ON) adhering to the spicule surface (note small spicules occurring below the net (**b)** and the ripple mark structures that occur in several layers). **c** Details of ripple mark structures occurring on the surface of several superimposed layers. **d** Cross section of several outer layers of the spicules with ripple mark structures that gradually disappear toward the spicule interior formed by PG

In some fragmentary material detached from a spicule, a fourth morphological structure was observed (Fig. 11) that is a regular striation of the surface of the PG layers. It resembles sedimentary ‘ripple-marks’, thus it is here called the ripple marks layer (RML). On this surface the particular striae are wavy and may be divided or fused (Fig. 11c), but in general they are longitudinally arranged. Due to the fragmentary character of the sample, the precise location of the RML along the spicule length is not determined. However, it is from the upper part of the spicule situated more toward the spicule apex than the TL, as it was not observed in transverse sections of the lower part of the spicule. The RML surface occurs on the 10–15 most external layers of the spicule passing gradually into the PG layer (Fig. 11d). It is covered directly by the organic net (Fig. 11a, b, d) and smaller spicules may occur between the net and surface of the basal spicule.

#### Organic material associated with the spicules

**Fig. 12:**
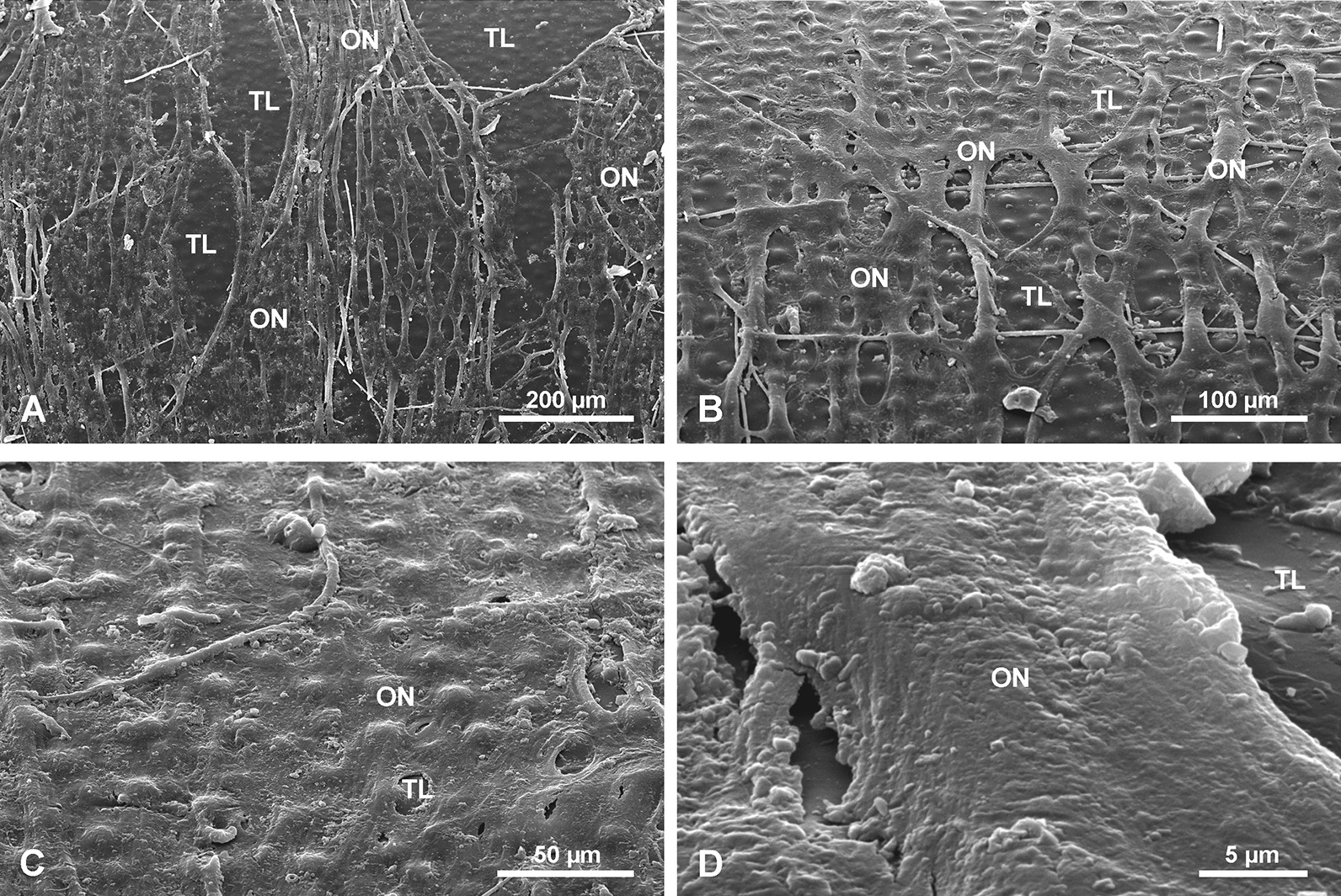
Organic net (ON) adhering to the surface (here shown above tuberculate layer surface). **a** General view. **b**, **c** Details showing organic net and its relation to incipient tubercles; note irregular meshes of the net and small spicules occurring below the net (**b)**. **d** Details of organic net showing its fibrous nature

Most of the investigated spicules were devoid of sponge organic matter but some had remnants of organic material attached to the surface of the spicules. A single complete specimen with a sponge body was examined. In this well-preserved specimen, a basal spicule is surrounded by a dense organic net (ON) (Fig. 12) with irregular, elongated, oval meshes of different size and shape that reach 70.9 to 101.5 µm in length and 21.7 to 56.6 µm in width for the largest meshes. Size and shape of meshes may differ from the living sponge because the specimens were dry. This organic net occurs above all morphological zones i.e., smooth PG zone, the TL zone, and the AL zone of *Monorhaphis* spicules. The meshes of the net do not correspond to tubercles of the TL surface, neither in size nor in shape, and the net may cover them completely, or several tubercles may correspond to one mesh (Fig. 12b, c). Smaller, thin monaxonic spicules often occur between the net and the basal spicule (Figs. 11a, b, 12b).

**Fig. 13:**
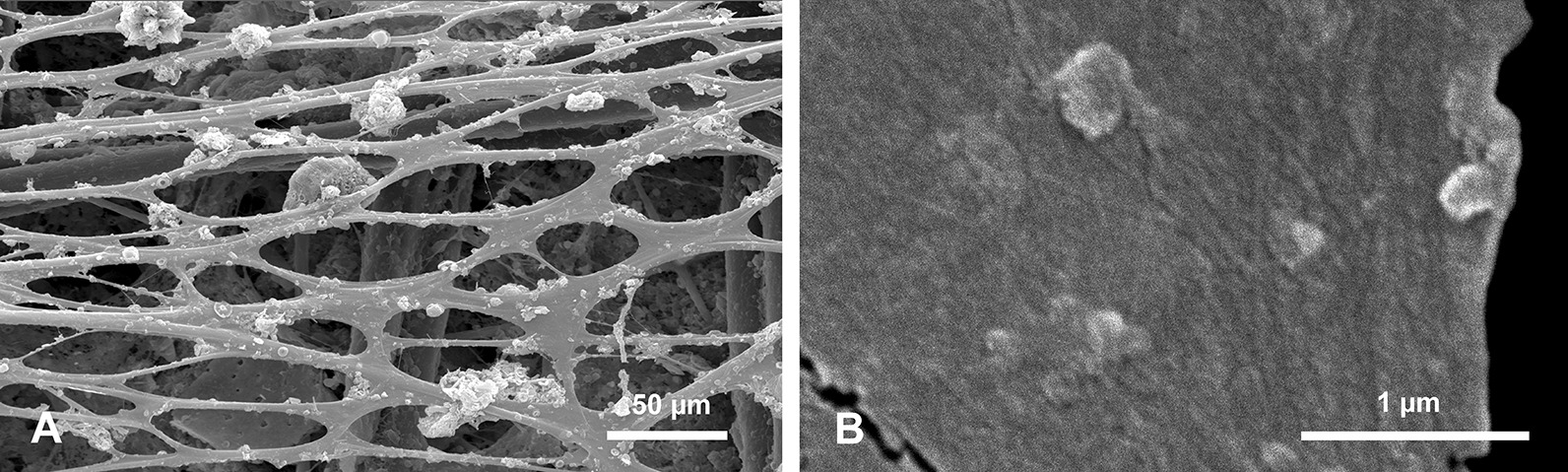
Organic (collagen) net (surface facing the basal spicule) detached from the spicules (bound together with the sponge body and other smaller spicules). **a** General view – smaller body spicules seen in meshes below the net. **b** Details of the net showing its fine fibrous structure

In complete specimens the organic material could be detached with the net intact, and the lower surface that adheres to the spicule surface could be examined (Fig. 13a). Under high magnification the fibrous structure of the organic material comprising the net was observed (Fig. 13b). Staining of this net with Sirius Red causes red coloration indicating that it is probably collagen [57], which agrees with the fibrous nature of the net.

**Fig. 14:**
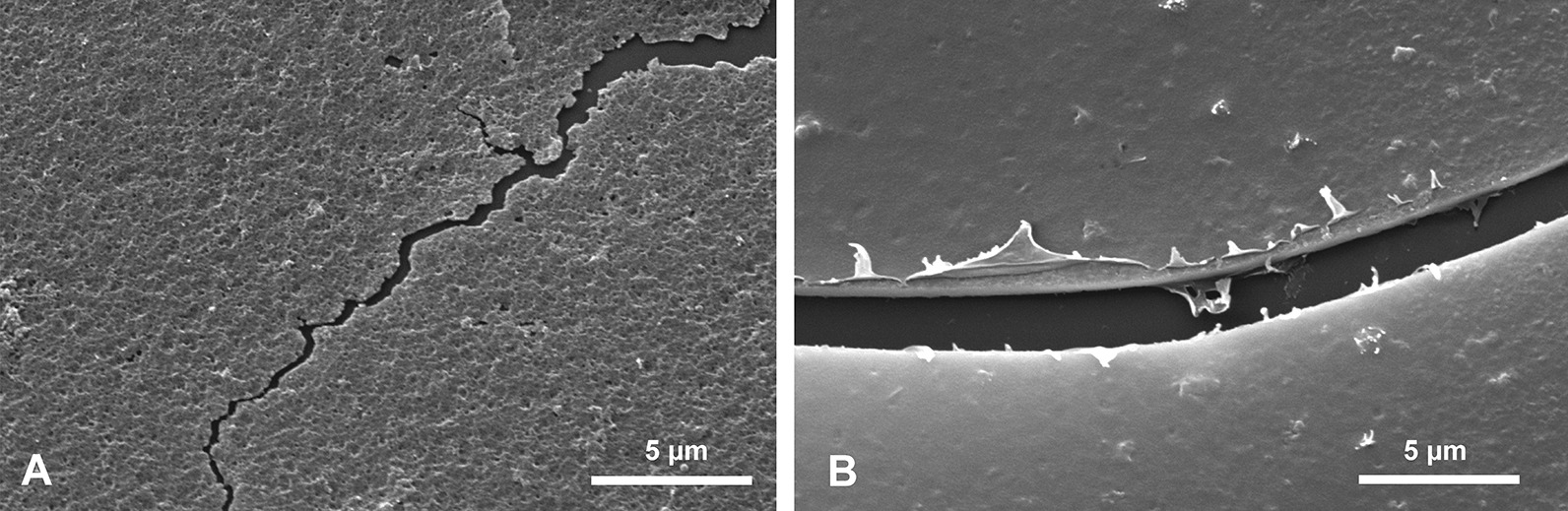
Fine granular structure of silica (**a**) and organic matter permeating the silica (**b**), visible after NaOH treatment. It covers the etched surface and is visible at broken edge of silica layer

Dissolution of the spicule fragments in 2.5 M solution of NaOH over several days (temperature 36º C) revealed a fine granular structure of silica (Fig. 14a) in all layers (finer than in the outermost AL). Organic material was also observed on etched surfaces and in fissures, as a thin organic film (Fig. 14b). This, most probably, corresponds to the brown hues of t he spicule visible in transmitted light and to various epifluorescence zones.

### NMR analysis

#### ^13^C CP-MAS ssNMR experiments (Fig. 15a)

**Fig. 15:**
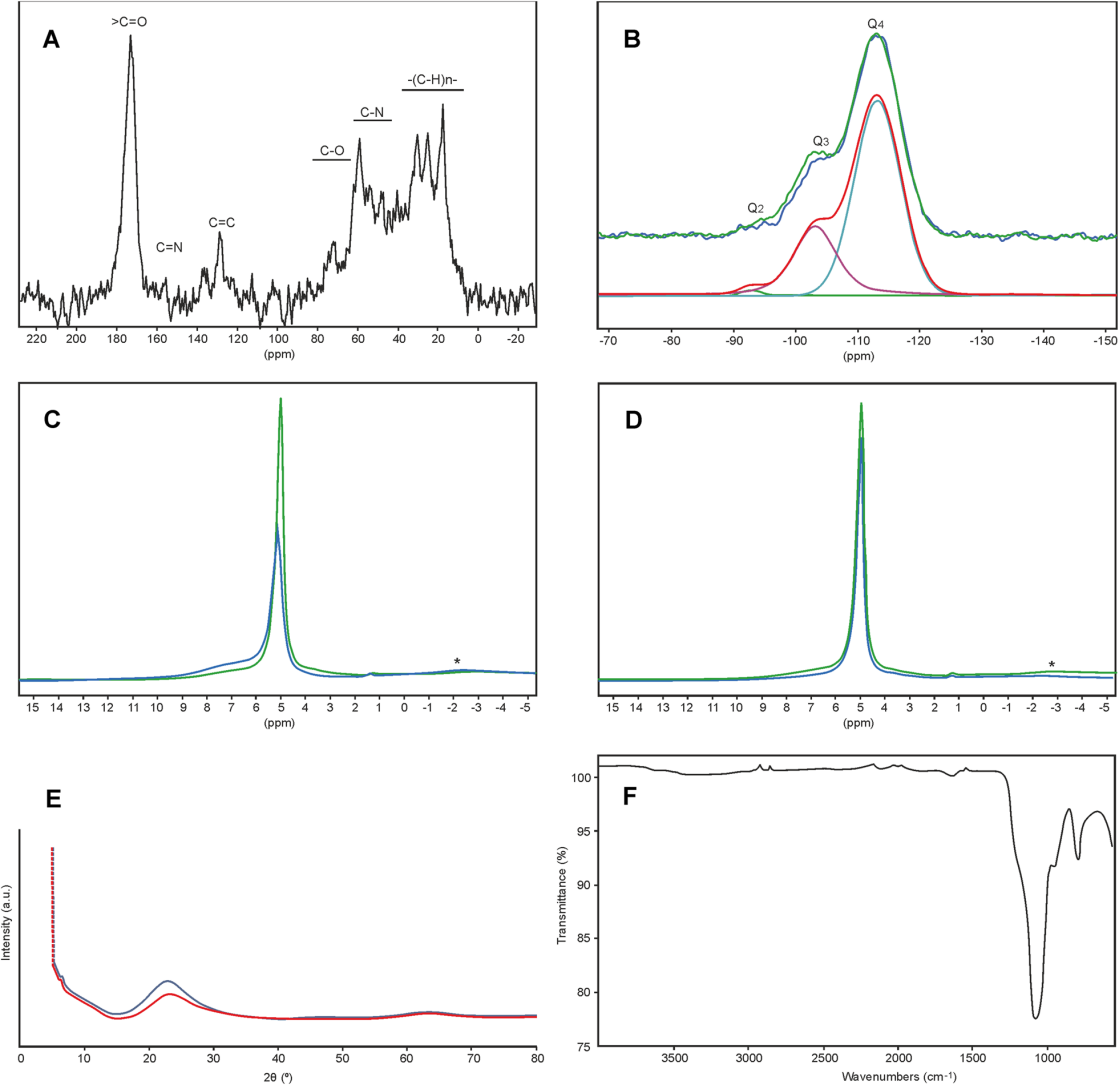
Graphs showing results of NMR, XRD and FTIR analyses. **a** ^13^C CP-MAS ssNMR spectrum of PG recorded on a Bruker AV300 spectrometer using a 4 mm CP-MAS Bruker probe at RO = 11.962 Hz (MAS), SF = 75.51 MHz, RD = 1 s, t_cp_ = 1 ms, collecting NS = 976.896 scans and using LB = 50 Hz for apodisation. **b** ^29^Si HPDec-MAS ssNMR spectra of AL (in blue) and PG (in green) recorded on a Bruker AV700 spectrometer using a 2.5 mm MAS Bruker probe at RO = 20 kHz (MAS), SF = 139.10 MHz, RD = 60 s at 30° pulse angle, collecting NS = 6.000 scans for sample AL (I) and 4.000 scans for PG spectrum, and using a line broadening LB = 100 Hz for the post-treatment. The spectral deconvolution using Dmfit software is shown for the AL underneath the spectra allowing calculation of Q_2_, Q_3_ and Q_4_ contributions. Spectra were normalized based on the Q_4_ component as the rotor could not be completely filled (approximately at two thirds), by lack of material. **c** ^1^H OnePulse MAS ssNMR spectra of AL (in blue) and PG (in green) recorded on a Bruker AV700 spectrometer using a 2.5 mm MAS Bruker probe at RO = 20 kHz, SF = 700.14 MHz, RD = 5 s with NS = 4 scans for both samples. For the purpose of a quantitative comparison of the spectral profiles, PG spectrum was drawn with a diminishing scaling factor (0.67), as the rotor of sample AL was filled only to 2/3. *: 2.5 BL probehead signal (systematically present, whatever the sample). **d** ^1^H MAS ssNMR spectra of glassy silica: Spin echo experiment (in blue) and OnePulse experiment (in green). Experiments were recorded on a Bruker AV700 spectrometer using a 2.5 mm MAS Bruker probe at RO = 20 kHz (MAS), SF = 700.14 MHz, RD = 5 s with NS = 16 scans for both. *2.5 BL probehead signal (systematically present, whatever the sample). **e** XRD powder diffractograms of AL (in black) and PG (in red). **f** FT-IR spectrum of the PG

The ^13^C {^1^H} CP MAS NMR spectrum of the PG layer is presented in Fig. 15a. The 0–40 ppm region is characterized by a broad band resulting from the overlap of numerous resonances that can be attributed to the aliphatic CH_x_ fragments found in proteins, carbohydrates or glycoproteins. The multiple resonances detected in the 40–60 ppm chemical shift region indicate the presence of C–N bonds, which possibly arise from protein chains and may be found, for example, in collagen. The 60–80 ppm region is more likely to correspond to C–O bonds usually found in carbohydrates, polysaccharides or in the oligosidic part of glycoproteins. Of note is the absence of signal (usually expected at 101 ppm) for the atypical C1 atom of carbohydrates, which is probably indicative of the glycosylation of the oligosidic parts that occurs during glycoprotein formation. In contrast, easily distinguished peaks can be found in the low-field region. The 129 ppm resonance corresponds to C=C double bonds that may originate from unsaturated fatty acids. The high-field resonance at 173 ppm is probably from the carbonyl resonance of amide groups that may be found in proteins [58]. They may originate from collagen, silicatein or copolypeptides [59], or glycoproteins such as lectines [36, 60], and even more specifically from galectins [61].

#### ^29^Si HPDec-MAS ssNMR experiments (Fig. 15b)

The ^29^Si ssNMR experiments were carried out using nearly quantitative conditions, routinely well-adapted to amorphous silica gel-like material observation, as expected for such siliceous skeletons. This quantification was then realized using DmFit software for spectral deconvolution [54]. The ^29^Si NMR spectra of the two samples – the PG and AL zones (Fig. 15b) – show three main broad lines attributed to Q_2,_ Q_3_ and Q_4_ species (i.e. [SiO_4_^4−^] silicate units called Q_n_ in which the considered Si atom is linked to n = 2, 3 or 4 oxygen-bonded silicon neighbors, respectively). The chemical shifts of these Q_n_ species are positioned at ca. δ = -93 ppm, − 103 ppm and − 113 ppm for Q_2,_ Q_3_ and Q_4_ species, respectively. The spectral deconvolution allows for calculation of the proportions of these three components, as gathered in Table 2, made on spectra with a similar signal/noise ratio. The silica condensation degree obtained is representative of an overall well-condensed silica network, in agreement with that was usually obtained for such siliceous sponge material [17, 62]. More precisely, D was assessed as 0.92 for the AL and 0.91 for the PG. That also means that, at a second level, the AL is slightly more condensed than the PG. There is less Q_2_ and Q_3_ but more Q_4_ in the AL, leading to an average connectivity degree $\overline{n}$ of 3.70 for the AL over 3.66 for the PG. This small difference of four hundredths is indicative of a subtle differentiation between the two samples.

**Table 2:** Results obtained after DmFit deconvolution of ^29^Si HPDec MAS ssNMR spectra of *Monorhaphis chuni,* AL and PG

Sample	Q_2_ (%, ± 1%)	Q_3_ (%, ± 1%)	Q_4_ (%, ± 1%)	D (± 0.01%)	$\overline{n}$(± 0.0 1%)
AL	0.9	28.3	70.8	0.92	3.70
PG	1.3	31.7	67.0	0.91	3.66

#### ^1^H MAS ssNMR experiments (Fig. 15c, d)

The ^1^H MAS ssNMR experiments were carried out using OnePulse and Hahn echo (Spin Echo) coupled to MAS at 20 kHz. Due to the small amount of the AL, the corresponding 2.5 mm rotor was not completely filled (approximately two thirds). For the purpose of the study, spectral intensity of PG signal was proportionaly reduced to compare the spectra (Fig. 15c). This technique allows assessment of the relative amount of the constitutive species found in the samples. Two peaks dominate the spectra that can be assigned to protons coming from a water peak (at ca. δ = 5.1 ppm) and protons coming from –OH (including silanols, Si–OH species) and –NH groups (much broader peak at δ ≈ 7 ppm). Minor peaks (traces) at ca. δ = 1.3 ppm and 3.8 ppm are also visible, that can probably be assigned to protons coming from –CH_3_ and –CH_2_- groups, respectively, as can be found in aliphatic chains in organic matter. The PG contains approximately twice the amount of protons from water than the AL. An advanced deconvolution of the spectra providing absolute data would not be realistic, but the comparison of the balance between the two components of the spectra could be considered. In particular, it is clear that the AL contains many more protons assigned to –OH and –NH species and less protons from water, compared to the PG.

Spin Echo experiments, that are representative of the most mobile species present on the spectrum of PG (Fig. 15d) show that the obtained echo peak represents the major part of the whole peak at 5.1 ppm, obtained by the former OnePulse experiment. Taking into account the relative width of this peak, it is possible to consider that this mobile component may correspond to adsorbed water, mainly constituted of water molecules physically adsorbed on the surface through H bonding. Other protons, corresponding to –OH and –NH groups, giving rise to the very broad peak centered at 7 ppm on the OnePulse spectrum, are less present on the Spin Echo spectrum, meaning that their mobility is very limited. Minor peaks at ca. δ = 1.3 ppm and 3.8 ppm are still visible in a very small amount (traces).

### XRD analysis (Fig. 15e)

A background recording was undertaken to distinguish precisely between baseline, amorphous and crystalline phases. The main signal could be assigned to the diffuse signal of an amorphous form of silica, more precisely an opaline-like silica phase, giving rise to a typical bump ranging from ca. 15° to 35° (2θ) (Fig. 15e). Supplementary minor peaks are also present on the diffractograms, whose definition is probably noticeably overvalued due to the use of the low background silicon sample holder. They may correspond to very well-crystallized phases not yet perfectly assigned, that could probably come from impurity grains or precipitates of sea water. Another minor peak (shoulder) at ca. 6.5° (2θ) is also visible, but does not seem to be linked with any known phase in this system and is likely an artefact.

Both experiments were undertaken in the same conditions, analysing precisely the same amount of powder (11.0 mg), that makes a quantitative comparison possible between both samples. Using data processing software (Fullprof), and after a baseline correction, the integrated intensity values show that the AL contains ca. 26% amorphous silica more than the PG. A separate and complementary experiment was conducted in the same conditions, using a pure amorphous silica specimen as a reference, to quantify the relative amount of silica in each sample. The relative integrated intensity of each sample, and the related relative uncertainty over the pure amorphous silica reference, were calculated, showing that AL and PG contain 69% (± 2%) and 50% (± 2%) of amorphous silica, respectively.

### FTIR analysis (Fig. 15f)

The FTIR spectrum of the PG is mainly dominated by specific vibrations of silicates (Fig. 15f). Their anti-symmetric stretching band Ʋ_as_, which corresponds to an active mode in IR, gives rise to a triply degenerated band. This very strong three-component vibration spreads over a very wide range, from 850 cm^−1^ to 1200 cm^−1^. In the case of highly-condensed silica such as in the *Monorhaphis* spicules, this wide band is mainly centered around 1100 cm^−1^, which can be seen in the spectrum with 3 vibrations at 955 cm^−1^ (m), 1073 cm^−1^ (vs), plus a shoulder at 1193 cm^−1^ (w). The corresponding symmetrical stretching band Ʋ_s_, despite being inactive in IR, gives rise to a band at 792 cm^−1^ (m). A complementary deformation vibration δ, is also visible at 551 cm^−1^ (vw).

A wide but very weak vibration, visible at 1622 cm^−1^ (vw), may correspond to the C=O vibration of amide groups in proteins, that could be related to additional minor bands at 2923 cm^−1^ (vw) and 2913 cm^−1^ (vw), for the conjugated N–H bonds.

## Discussion

Most of the structures described above for *Monorhaphis* basal spicules were observed and described by Schulze [9] at the level possible for that time. Recently, Müller et al. [25] and Wang et al. [34–36, 38, 39, with references] presented descriptions of the same structures and proposed a model for spicule morphogenesis. We compare and contrast our examination of the structures of these spicules with those in earlier studies.

### Organic material associated with basal Monorhaphis spicules

Our observations of organic material occurring on the surface of the *Monorhaphis* spicules conforms with earlier reports by Schulze [9]. He described “*der faserigen Nadelscheide*” [fibrous sheath of spicule] in smaller body spicules [9: Pl. XL, fig. 7] and “*Netz der faserigen Nadelscheide*” [net of fibrous sheath of spicule] in the mature basal spicule [9: Pl. XL fig. 8]. The collagen nature of this net was recognized by Ehrlich et al. [63, 64] who also demonstrated that it is hydroxylated fibrillary collagen [65]. The fact that it can be easily detached from the basal spicule surface, that small body spicules occur between the net (having fibrous structure—Figs. 12d, 13b) and the basal spicule surface (Figs. 11b, 12b, 13a), and that there is no direct relation between the meshes of the net and the tubercles and ridges on the spicule surface, suggest that the net is not directly participating in the process of basal spicule biomineralization, which must instead be controlled by the sclerosyncytium (multinucleate scleroblast masses) [42]. In our opinion, the net is better considered as a structural element of the sponge body keeping it connected with the basal spicule.

Apart from the exterior collagen net that covers the spicule surface, organic substances permeate the spicule silica (Fig. 14b) and our analysis indicates that these are proteins which were already reported– silicatein and/or galectin [2, 28], or collagen [3, 64, 65]. The ability to precipitate silica by much more simple synthetic minicollagens has been demonstrated [66].

### Spicule structure

The nature of the axial filament of the studied spicules was beyond the scope of the present study, but there are several recent attempts to understand this in spicules of *Monorhaphis* and other sponges using X-ray based approaches with respect to silicateins [47–50]. However, no experimental evidence was presented in these publications for the presence of silicateins in the samples under study, but it was assumed, and there was no reported attempt to demineralize the spicule with the aim of isolating and characterizing the silicateins. Thus, the nature and origin of the axial filament in *Monorhaphis* remains to be determined.

**Fig. 16:**
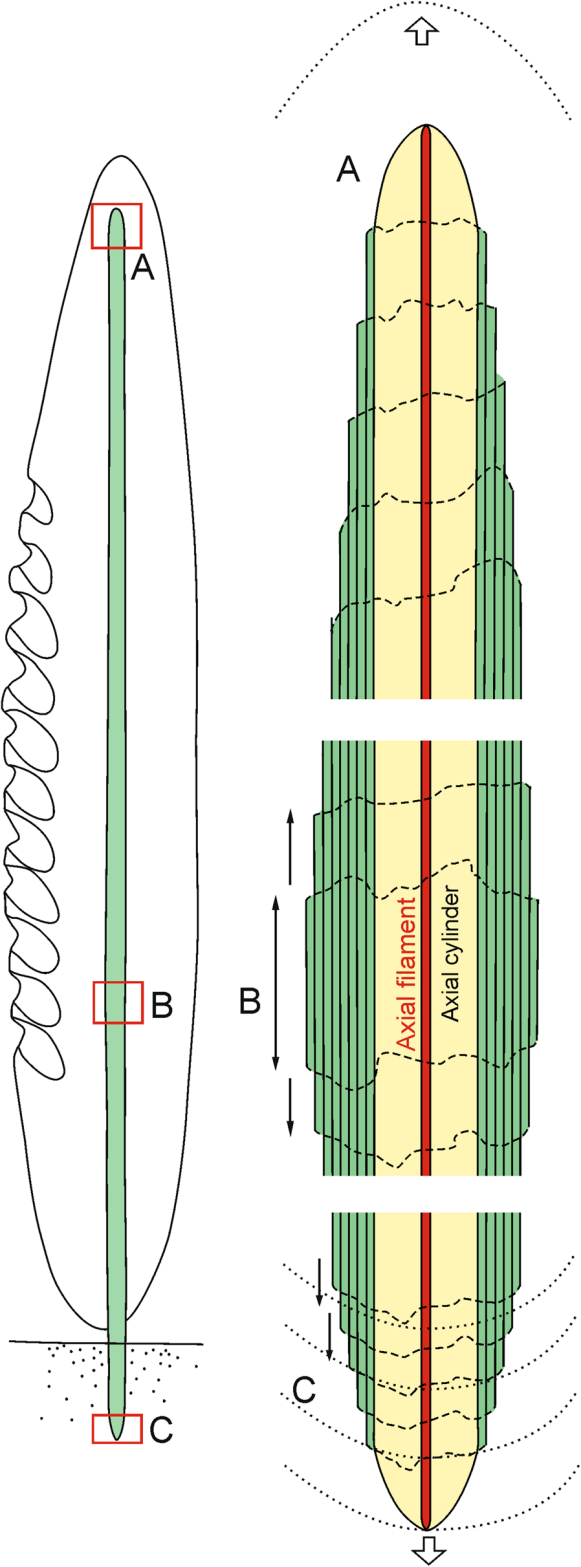
A model for early (young spicule) stages of growth of a basal spicule. **Left** relation between spicule and sponge body. **Right** details of various structural zones. Red–AF; yellow–AC; green–layered PG silica

**Fig. 17:**
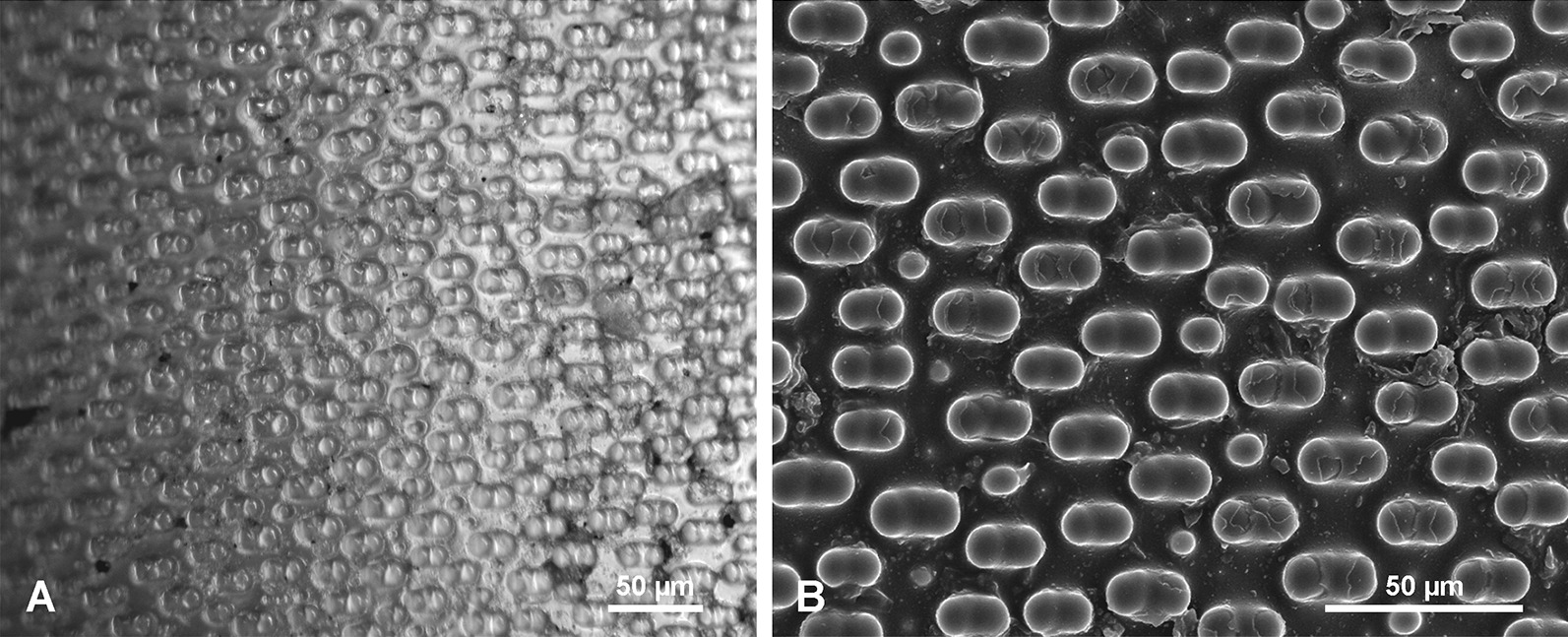
The same tuberculate surface as seen in reflected light (**a**) and in SEM (**b**). Tubercles in oblique reflected light give an impression of depressions

All earlier papers described very regularly layered structures of the basal spicules in transverse section [9, 17, 30, 38, 39, 48, with references] composed mainly of PG layers (Figs. 2, 3, 5). At the nanoscale the silica of the PG layers has a granular structure [25, 36, Fig. 14a] what is typical of other hexactinellids as well [14]. The material that builds the spicules is always amorphous opaline silica and differs slightly in structure between the outermost AL, which is more condensed than the rest of the spicule. This could be explained by a smaller amount of structural water in the amorphous silica of the AL than in the rest of the spicule (figs. 16, 17).

**Fig. 18:**
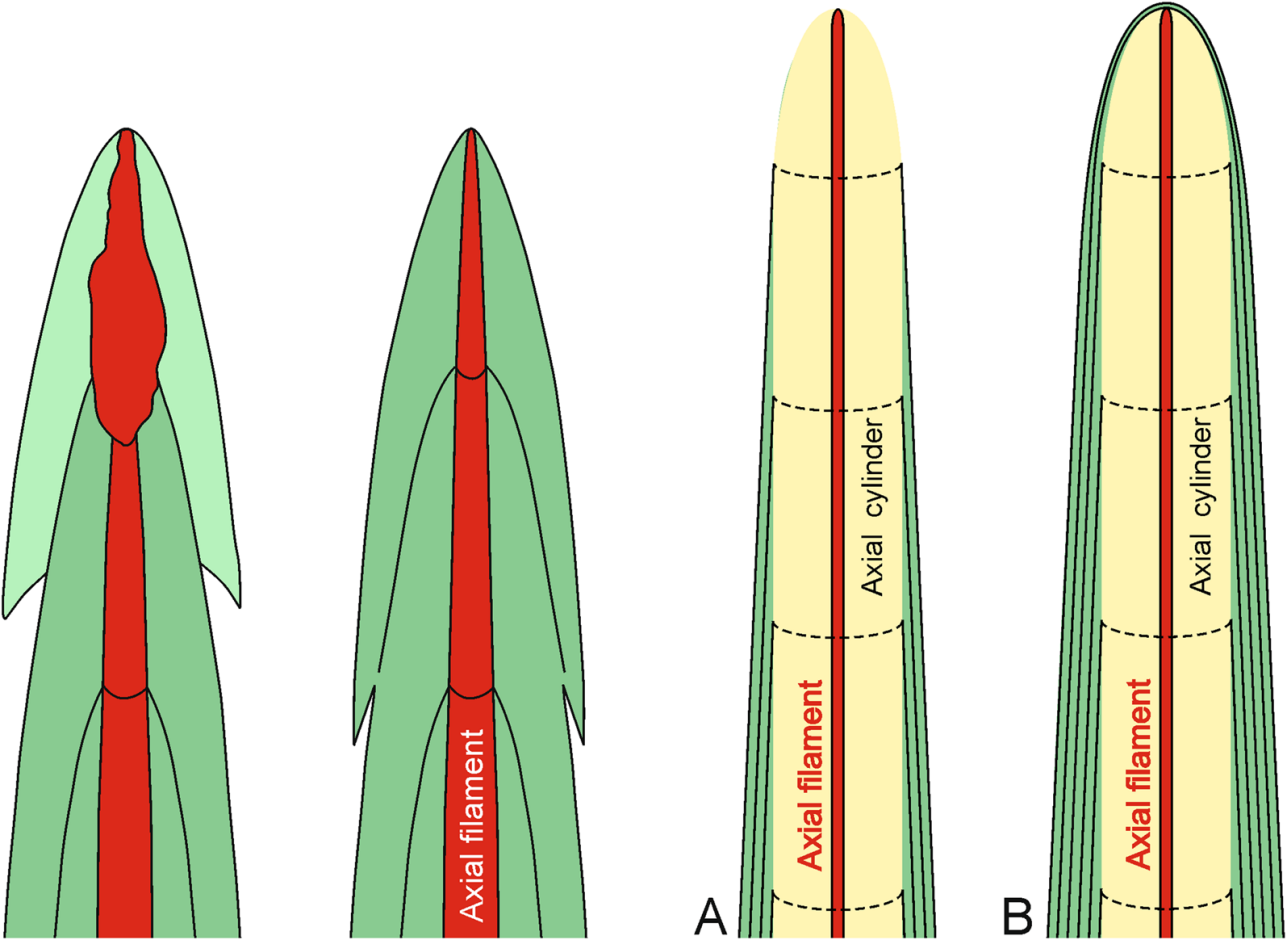
A model of spicule growth at the apical tip proposed by Wang [28, 29], modified (left) and as revealed by the present study (right). **a** Stage when spicule is still growing. **b** Final stage when growth is arrested. Red–AF, yellow–C, green–layered PG silica

Our studies of the immature spicule, which is characterized by the presence exclusively of PG layers (this corresponds to a growth stage illustrated by Schulze [9] when the sponge body has enveloped a major part of a spicule) lead us to conclude that they do not follow the growth model proposed by Wang et al. [24–36, 38, 39]. These authors used comitalia (smaller body spicules) of *Monorhaphis* as the basis of their reasoning, not a large basal spicule, but didn’t show that the structure of these spicules is the same as a basal spicule. In addition, they used an artificially broken spicule and the resultant detachment pattern of the apical spicule layers [34]. They reported step-like structures of broken layers (with steps facing downward, toward the spicule center) that were interpreted as mimicking the spicule growth pattern [34: fig. 5a, p. 277]. This result subsequently influenced interpretation and modelling of *Monorhaphis* spicule growth. They suggested that growth in spicule length proceeds by cones being added one above another from the apex of the spicule, which later extend downward (Fig. 18 left). Thus, new layers are added by a downward extension of the cones, and by engulfing already existing spicule layers (Fig. 18 left). Our observations of the young basal spicule clearly indicate that growth is fundamentally different, as discussed below (Figs. 18 right and 19).

**Fig. 19:**
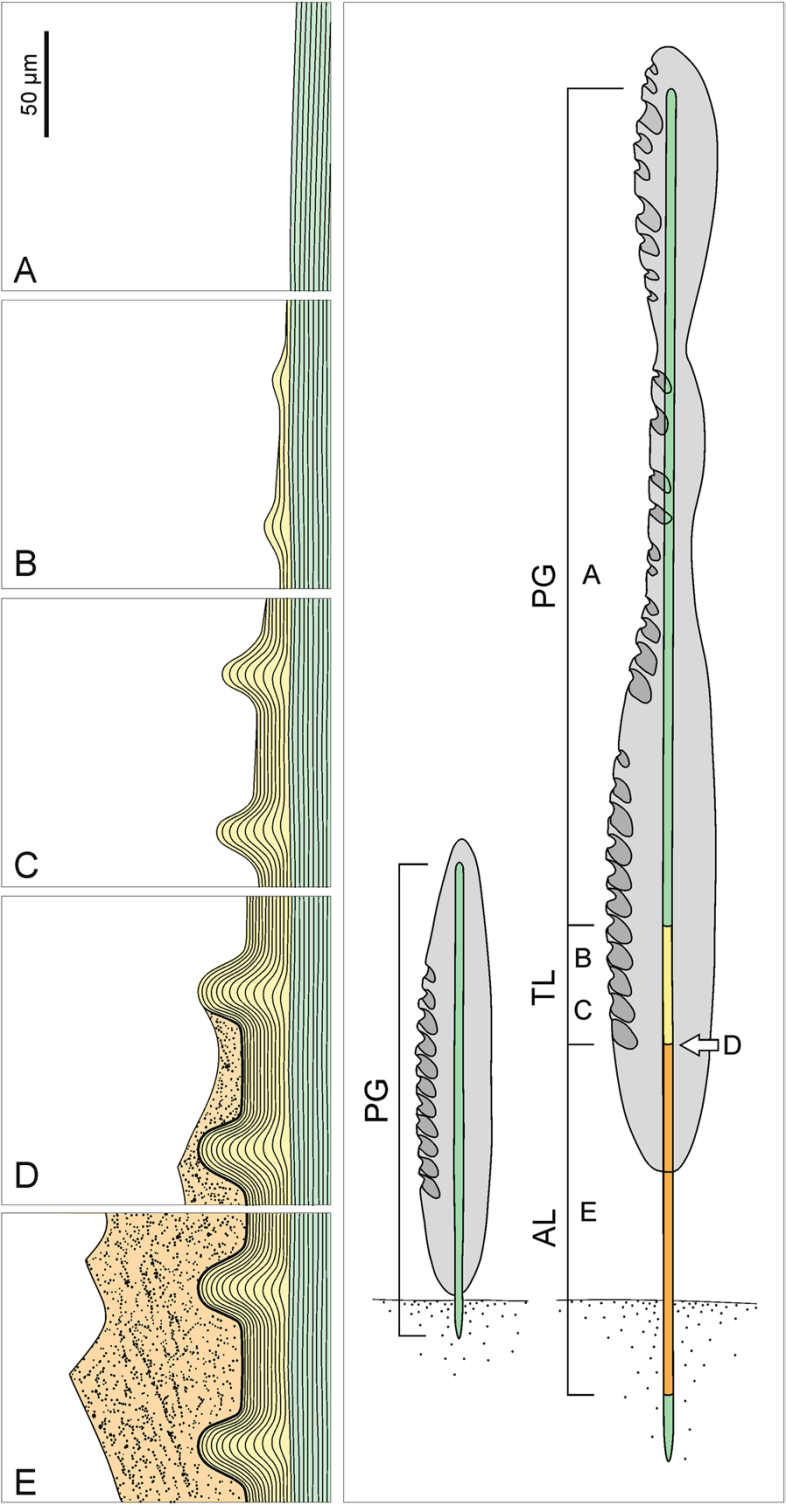
A model of structural organization and growth of the basal *Monorhaphis* spicule as revealed by this study. left Structural zones as seen in adult spicule: **A** PG (plain glassy) layers. **B** incipient TL (tuberculate) layer above PG. **C** advanced stage of TL. **D** Incipient AL (annular layer) above TL. **E** Fully developed AL above TL. middle Young sponge with spicule consisting only of PG layers (green); sponge body (grey) and sediment (dotted). Right: adult sponge showing various structural zones [green–PG (plain glassy silica) zone, yellow–TL (tuberculate layer) zone, orange–AL (annular layer) zone] and their relation to the sponge body (grey). A, B, C, D, E correspond to the boxes on the left figure. Sediment dotted

The natural surface of the young spicule and its well preserved apex showed the presence of more or less natural step-like faces directed toward the spicule tips (not the center) and we suggest these correspond to growth lines. These lines or steps are more pronounced and more densely spaced near the lower tip of the spicule, and less well-pronounced and more widely spaced at the apex. This indicates that growth or extension of the spicule is proceeding in the direction of the tips (Fig. 18 right), not from the upper tip toward the center. Moreover, it suggests that growth of different layers (PG, TL and AL) may proceed simultaneously in different locations along the spicule. In other words, formation of one layer does not proceed over the whole spicule length and surface immediately, but initially is spatially limited. Internal layers may lengthen closer to the upper tip, while thickening of the spicules is achieved by progressive growth of more external layers. We also observed that the faces of growth lines in the center of the spicule are in opposite directions in the young spicule (Fig. 2c, h). Those in the upper portion of the spicule show step-like faces directed upward (Fig. 1a, b), while those from the lower part are directed downward (Fig. 2d–f), thus, the initial growth of the spicule must be bidirectional, as could be expected from what we know about smaller hexactinellid monaxial spicules (diactines).

The interpretation of the growth of the *Monorhaphis* basal spicule proposed in this study is also supported from a biological viewpoint, because *Monorhaphis*, as for all hexactinellid sponges, is syncytial in nature, and thus silica may be deposited in any location on the spicule under the body cover. Conversely the model of Wang et al. [34–36, 38, 39] suggests that growth occurs only at the upper tip of the basal spicule (and thus also of the sponge). Our observations are in contrast to the “cone in cone structure” model [34–36, 38, 39] and in agreement with Schulze’s [9] observations. He illustrated specimens with the upper portion of the spicule protruding from the sponge body. Such a situation is not possible in the model by Wang et al. [34–36, 38, 39], but entirely plausible in the light of our observations, hence our new proposed growth model.

The simultaneous lengthwise growth of the spicules in both directions characterizes only the early stages of spicule development when it is nearly completely covered by a sponge body (Fig. 16). When the lower tip is well inserted into sediment, the sponge body is “climbing up” the spicule [9]. This movement of the sponge body from the lower part of the spicule upwards is registered by showing step-like faces of more external layers directed toward the lower spicule tip (Figs. 2d–f and 18), while growth at the upper tip progresses upward.

#### Tuberculate layer (TL)

The tuberculate structure was previously illustrated and described [9: Pl. XLIV, figs. 1–3, 8]. It has also been described in several recent papers [25, 34], but there are some inconsistencies in these descriptions [25: fig. 3f]. It was reported that the surface of the basal spicule is “*occasionally decorated by rectangular protrusions*” [25: fig. 3f]. Some suggested [34: fig. 3c, d] that a collagen net with holes and tubercles [“hemi-spherical” protrusions (knobs)] and that the protrusions (= tubercles) fit into the holes of the collagen net and are of similar size. However, their observations were not on basal spicules but much smaller body spicules, that are smooth. Subsequently, we have demonstrated that there is no direct relationship between the net openings and the tubercles (Fig. 12). Additionally, the shape of the holes in the net do not correspond to the shape of the tubercles, disposition of the holes in the net is irregular and different from the disposition of the tubercles on the spicule surface (see Figs. 12, 13) which show high regularity. Thus, the suggestion that tubercles (= protrusion) “*exist in the initial phase of silica formation*” [34] has not been confirmed in this study, and neither has the suggestion that *“At later stages, the protrusions seem to melt”.* The growth of tubercles in size and change of shape is gradual and it is the second (the first being PG) phase of silica deposition. At later stages they are gradually covered by incipient AL (Fig. 6).

According to Wang et al. [39: p. 2050, explanation to fig. 2], the zone of smooth surface is followed by *„zone of tiny coarse protrusions (tc)*” or that “*penultimate lamellae* [is] *cluttered with tiny bumps*” [and]”*a thin layer of a finely woven network (fn)”* that under higher magnification is revealed as “*layer riddled with depressions with elevated rims (fig. 2e, i)*”. We have shown with SEM observation (Fig. 6e, f), “protrusions” pass gradually into fully developed protrusions/tubercles and those, in turn, pass gradually into (are covered by) the AL (Fig. 6b–d). The apparent depressions [39] are in fact an optical artefact caused by specific illumination of the surface with tubercles (see Fig. 10e). The same surface under oblique reflected light looks like it has depressions (optical illusion) and with SEM categorically shows tubercles (Fig. 17).

#### Annular layer (AL)

As demonstrated by our analyses, the AL layer is of mineral opaline composition, is similar to the rest of the spicule but differing in structure. It corresponds to the “*Querriffle zone*” [transverse hackle zone] of Schulze [9: p. 118] who also considered it to be mineral, and the “banded ribbon” zone of others [34, 35, 39], who first suggested that it is a “*collagen net*” [34: p. 273, fig. 3c, d, 35: fig. 5b]. We propose to call it the “annular layer”, because the name proposed earlier [39] is based partly on the presence of “bands” that are taphonomic or optical artefacts, not original features (see below). Wang et al. [35: fig. 3.10c] also marked and described the AL as “*a solid fibrous (collagen) sheet (fi)*” on a copy of the original figure from Schulze [9: Pl. XLIV, fig. 8a], where it is clearly described as “*Querriffel ziegenden Lamellen*” [(hackle with lamellae); fig. 8a, p. 119].

The darker/grey and milky bands observed in our study on the surface of the AL under reflected light (but not with SEM; Fig. 10), correspond, without doubt, to the bands described earlier [39: p. 2051). It is suggested there that there is regularity in the width and distribution of these bands, and that there are two types: non-perforated and perforated [39: fig. 5b]. In contrast, our study found no regularity in the bands, and no perforated or non-perforated ones (Fig. 10). The AL is a solid silica layer with a granular structure and high organic content along its entire length. This can also be seen in figure 5a in Wang et al. [39].

These bands are not visible with SEM (Fig. 10b) and the difference in colour results from the fact that the lighter (milky) bands are not bound perfectly with the underlying TL; there is a micro-fissure between the AL and the TL surface, while the darker bands are tightly bound with the underlying TL. As a result of light refraction at the boundary between the AL and TL, when a fissure exists between the two layers, an impression of different colours of the bands appears. In the case of the darker bands, there is no light refraction at the boundary with the underlying TL because there is no fissure between the layers. Consequently they behave as an optically homogenous structure. The “*depressions*” or “*perforations*” [39: figs 2l, 5b–d, f, g] occurring in the lighter bands of the AL are an optical artefact corresponding to the tubercles (protrusions) of the underlying TL as seen through the silica of the AL. This taphonomic feature could occur after sponge death, i.e. during spicule collection, by a mechanical stress that can partly detach the AL from underlying TL surface. However, it cannot be excluded that this may have happened in earlier stages when the sponge was still alive, and it is due to differential stress caused by bending of the spicule in various directions when it was still anchored in the sediment.

An additional point that challenges interpretation of apparent depressions as locations of sclerocytes is the fact that hexactinellids only have sclerosyncytium [42], and we consider that this syncytial nature allows for formation of large siliceous structures such as the basal spicules of *Monorhaphis*, that would be not possible in cellular sponges [cf, 16] It is worth noting that the size of the megascleres in syncytial hexactinellids, are in general larger than the size of megascleres in demosponges, which are cellular.

A complex scenario of deposition of siliceous lamellae by supposed discrete sclerocytes located in the “*depressions*” on the spicule surface (despite the syncytial nature of hexactinellids) was developed [34, 35, 39] and claimed without evidence that there is “*no reason to believe that all cells participating in lamella formation fuse to form syncytia*” [39: p. 2053].

It was shown [35] that the axial cylinder contains proteinaceous material and called it the axial barrel, and its careful examination in transmitted light reveals that the axial cylinder is layered (however not regularly) (Fig. 5h, i), also shown by Schulze [9] and reaffirmed by Wang et al. [35]. The apparently homogenous structure of the axial cylinder, visible on a broken surface with SEM (Fig. 2k) shows a difference in organic content, resulting in different mechanical properties during the spicule breaking. Our observations demonstrate that there is a gradual transition from the “axial cylinder” toward the more external layered part of the spicule, that has lower organic content indicated by the absence of a brown hue (Fig. 5i).

Based on our observations and the discussion of ideas presented by Wang et al. [34–36, 38, 39] we propose a new model of *Monorhaphis* basal spicule morphogenesis.

### The new model of basal *Monorhaphis* spicule structure and morphogenesis

After careful consideration of our observations and relevant literature, especially those of Schulze [9], we propose the following growth model for the basal spicule of *M. chuni*, which includes the various structural zones and their functional interpretations.

The early stages of spicule formation, when most of the spicule is covered by a soft organic body is rather rapid in relation to later growth stage, and increase in length proceeds in two directions, towards both tips of the spicule at unequal speed (Figs. 18, 19 middle). This is seen by the asymmetry of the spicule ends, the lower one being widely conical (see Fig. 2d–f), and the upper one being thinner and more narrow conical (Fig. 2a, b) suggesting faster growth toward the spicule apex. The direction of growth is maintained by the extension of the axial filament whose tip is not enclosed by silica during most of the process. It is not clear if it is ever enclosed by silica layer(s) in the final stage (Fig. 18 right: B) as has been observed for smaller spicules of *Monorhaphis*, and in demosponge spicules (Pisera unpublished).

The formation of axial filament and incipient silica layer in demosponges that are cellular and have discrete sclrocytes, is first intracellular after, during extracellular stage [17, 40, with references] axial filament is still growing for a limited time, but finally it is encased completely by several silica layers (Pisera unpublished observations). This terminates, in most cases, spicule elongation/enlargement (some exceptions to this scheme, such as desmas and large spicules of *Thenea,* that still are not well understood). However, hexactinellids are syncytial and have sclerosyncytium, and thus continuous growth (including axial filament growth that might be not encased by silica at all) may be maintained for a very long time, producing much larger spicules.

The ‘axial cylinder’ in *Monorhaphis* is formed next, and is already under the influence of the sclerosyncytium, not the axial filament. The axial cylinder occurs along the entire length of the spicule (Figs. 17, 18 right: A, B), hence its formation, as for the extension of the axial filament, must be continuous and simultaneous with the deposition of outer, layered silica in older parts of the spicule.

In the next step, PG layers begin to be deposited, which allows for an increase in spicule thickness. Some surface layers seem to be discontinuous initially. New layers begin to be formed in the central part of the spicule (Figs. 2c, h, 18) and are later extended toward the spicule tips. It appears that deposition of silica may be patchy, and that these patches are gradually fused into one continuous layer; each new layer transgressing over the earlier layer occurring below and near the tip (Fig. 18 right: A, B). The pattern of growth at the lower and apical tips differs. At the lower tip each successive (more external) layer has a reduced downward extent (layers are regressive in relation to underlying layers) (Fig. 2d–f). This may be because the sponge body is moving up the spicule (or the spicule is extruded downward), leaving the lower tip more and more exposed, and thus allowing for anchoring the sponge in the sediment (Fig. 18 left and 19 middle and right).

Additional growth results in formation of other morphologically and structurally various zones (Fig. 19) on the spicule surface. In the upper part of the spicule additional PG layers are formed (Fig. 19 left), while in the lower part two new structures appear: first tuberculation (Fig. 19 left: B, C) of the surface, and then (both in time and space), the AL (Fig. 19 left: D, E). The tubercles are formed by adding folded PG layers. Structurally TL is the same as a continuation of the PG. Tubercles are developed by progressive growth (down the spicule) in size and change in shape, by adding new, more folded layers above those beneath (Fig. 19 left: B, C).

After the tubercles reach their final size and shape, an incipient AL zone progressively starts to cover the TL (Fig. 19 left: D). The AL is structurally different from both the PG and TL by being granular and containing more organic material. The AL is always downward from the TL, and above fully formed tubercles. It appears that the AL is developed when the sponge body “climbs up” the spicule to be separated from the muddy sediment. There is no doubt that the AL is partly covered by sponge body (confirmed by direct observation) during its formation, but in lower parts is devoid of organic material even when the sponge is alive (Fig. 19 right), thus, no further growth in thickness takes place. During the later stages of spicule growth, the pattern of silica deposition differs in various zones and proceeds differently from the early stages of growth. Lengthwise spicule growth only takes place at the apical tip by the addition of new layers of PG which grow over previous layers (Fig. 18 right). This also occurs in young spicules which produce the PG. The growth at the lower part is arrested from the moment the AL is fully developed. In fact, the AL is deposited in an upward direction as is the TL zone. These layers are added above the TL and PG zones respectively as new layers (Fig. 19 left: B, D), and over each other during the course of the sponge “climbing” the spicule, thus adding to the thickness of the spicule. The result is that the sponge body moves up by extending the spicule, the TL surface is “moving up” the spicule by development of new incipient tubercles, and the AL is also “moving up” progressively covering fully developed tubercles.

The distribution of various morphological zones and structures on the spicule surface confirms that they are formed simultaneously on different parts of the spicule. The syncytial nature of hexactinellids is responsible for this pattern, as the syncytium may be functionally differentiated [42].

It is not clear why the AL and TL developed, but one can speculate that the outer AL is an adaptation to assist the sponge to remain associated with the spicule surface when “climbing up” during growth and it also prevents the sponge from sliding downwards on what would otherwise be a very smooth silica surface. The structure where elevations of the TL fit into depressions of the lower surface of the AL (Fig. 9b, c), can be compared to structural joints in woodworking called tenon and mortise or dovetail joints, used to connect two pieces of material to obtain a strong stable joint. Such joints help to keep the spicule layers together, stabilize and strengthen them and make the whole structure less flexible. However, the upper part of the spicule built only with PG and smooth layers, allows for bending of the spicule due to the possibility of movements along the layer’s surface. We hypothesise that the function of such a structure is to strengthen the spicule to prevent breakage (or bending) in the lower part and thus to assure sponge survival by keeping it above the muddy sediment. Some additional rigidity is achieved in the upper part of the spicule (covered with the soft body) by the presence of numerous other spicules – tautactins (of different size and diameter) that are located close to the basal spicule with their long rays tangential to the basal spicule.

Finally, one should stress that the basal spicule of *Monorhaphis*, with its unique features (including its large size and complex morphology), cannot be used as a general model of siliceous spicule formation, or even as a model for other smaller hexactinellid spicules (even other smaller body spicules of *Monorhaphis*), except perhaps for other basal hexactinellid spicules (e.g. spicules of *Hyalonema* which have a similar function and display similar surface structures; Pisera, unpublished data). However, the basic principle of increase in spicule length is the same in hexactinellid spicules (as well as in demosponge spicules), and is toward the spicule tips, not from the tips toward the spicule centre.

## Conclusions

Basal spicules of the hexactinellid sponge species *Monorhaphis chuni* were comprehensively examined to document surface features, structure, and geochemical properties. The study revealed three morphologically and structurally different silica layers occurring on the surface: a plain glassy layer (PG), a tuberculate layer (TL), and an annular layer (AL) in adult spicules. In addition, a central core, called the axial cylinder (AC), previously believed to consist of homogeneous silica was found to be layered, as indicated by different coloration (Fig. 5h, i), and epifluorescence (Fig. 5c), and interpreted as variations in organics content Young, immature spicules contain only PG layers. In adult spicules both PG and TL structural zones were found to have internal layering. On the spicule surface the three layers replace each other along the spicule length. In older parts of the spicules the three layers are superimposed, with AL being the most external and occurring only in the lower part of the spicules and TL being intermediate between the AL and PG. The PG layer dominates the volume of the spicules and constitutes the core, but is visible on the surface only in the middle and upper parts of the spicules. This zone is composed of numerous concentric layers. The TL is composed of several thinner layers formed by a progressive folding of the surfaces of the layers and its microstructure is the same as the PG layer. The AL differs significantly from the PG and TL in being granular and porous in structure.

Previously noted perforated and non-perforated bands of the AL were found in this study to be an optical artefact, and the ornamentation of the TL was protruding tubercles in contrast to some earlier descriptions that treated them as “depressions”. In this study we found a previously undescribed fourth layer type we have named the Ripple Mark Layer (RML), as well as narrow spikes on the AL ridges, also not previously described.

The interface of the TL and AL consists of tubercles that fit into depressions in the lower AL surface, thus forming a structure that may be interpreted as tenon and mortise or dovetail joints. This structure results in very strong and stable joints that stabilize the layers and stiffen and strengthen the spicules ensuring they are less prone to breaking in the lower part.

As a result of ^29^Si HPDec-MAS ssNMR and ^1^H MAS ssNMR, FTIR and X-ray analyses we demonstrated that the spicules are composed of well condensed silica [cf. 17, 62] with the outermost AL characterized by slightly more condensed silica, with less water, than the rest of the spicule. Etching in NaOH revealed that the silica is permeated by homogenous organics of protein character, most probably collagen, silicatein and/or galectins, as indicated by ^13^C CP-MAS ssNMR analyses.

The increase in spicule length is controlled by extension of the apical part of the axial filament that is not enclosed by silica. The sponge sclerosyncytium controls the deposition of outer layers. In this study no spicule structures could be related to the presence of sclerocytes in *Monorhaphis*. The initial growth of basal spicules is bidirectional and proceeds from the spicule center to the spicule tips by adding new PG layers, which may be discontinuous in the early stages of formation. The relationships between the layers indicate that their growth may proceed simultaneously in different locations along the spicule: this is possible due to the syncytial nature of hexactinellid sponges. In the latter stages of growth, when the sponge “climbs” the spicule, the growth becomes unidirectional toward the spicule apex by extension of particular zones and the addition of PG layers, thus allowing for an increase in spicule diameter. Deposition of the AL terminates the spicule growth in diameter in the lower part while it continues to grow in length and thickness in the upper part. The AL with its ridges and spikes helps the sponge to “climb up” and to stay in a stable position on the otherwise smooth spicule. The external collagenous net that envelopes the basal spicule is a structural element keeping the sponge body bound to the basal spicule and does not control tubercle formation.

This new model of growth in *Monorhaphis* basal spicules contrasts with the previous model of “cone in cone” growth, which suggested growth occurred by the addition of new “cones” at the top of the spicule.

Due to its unique structure, which is related to its function as a large anchoring spicule, the basal spicule of *Monorhaphis chuni* cannot be treated as a reliable model for growth in other hexactinellid spicules.

## Data Availability

All study material will be deposited in the collection of the Institute of Paleobiology, Warsaw, Poland, under acronym ZPAL.
